# Supplementary material for: Do sex hormones at birth predict later-life economic preferences? Evidence from a pregnancy birth cohort study
Source: Proc Biol Sci. 2020 Dec 23;287(1941):20201756. doi: 10.1098/rspb.2020.1756 (PMC7779492; doi:10.1098/rspb.2020.1756)
Supplement: Supplementary materials [file rspb20201756supp1.pdf]

Supplementary Materials for:

Do sex hormones at birth predict later-life economic preferences? Evidence from a pregnancy birth cohort study

Boris van Leeuwen, Paul Smeets, Jeanne Bovet, Gideon Nave, Jonathan Stieglitz, Andrew Whitehouse

S1 Experimental and survey measures of economic preferences

The experiment consisted of five incentivized tasks and several survey questions. The five incentivized tasks form the basis of our measures of economic preferences, together with two survey questions.

We measured risk preferences in two ways, one of which is incentivized. The incentivized measure is based on the method of Eckel and Grossman (2002). Participants choose one of six lotteries, which progressively increase in their financial risk. The expected value increases for the first five lotteries as well, but decreases for the sixth lottery. Hence, risk averse participants will select one of the first four lotteries (depending on their level of risk aversion), risk neutral participants will select lottery #5 and risk seeking participants will select lottery #6. Hence, those selecting a riskier lottery are more risk tolerant. The self-reported measure is based on the validated question by Dohmen et al. (2011), which asks how willing people are to take risks in general. Participants answer using a 7-point Likert scale ranging from "completely unwilling to take risks" to "very willing to take risks" (whereas Dohmen et al. (2011) use a scale from 0 to 10).

To measure competitiveness, we used a version of the task developed by Niederle and Vesterlund (2007). We used the version developed by Buser et al. (2018) that was intended to be used online. In this version, participants solve 'math puzzles'. Each math puzzle is a matrix with eight different two-decimal numbers. Participants have to select the two numbers in the matrix that sum to 10. The task consists of two parts. In Part 1, participants solve puzzles for three minutes and are rewarded using a piece-rate scheme: they receive 10 AUD (7 US$) for each correctly solved puzzle. Part 1 provides a measure of ability, which we use as a control variable. Part 2 consists of another three minutes of solving puzzles. At the beginning of Part 2,

participants choose how they would like to be rewarded for Part 2: either a ‘piece rate’ payment (as in Part 1: 10 AUD (7 US$) per correct puzzle) or a ‘tournament’ payment. If they choose ‘tournament’, their Part 2 score will be compared with that of a randomly selected other participant (who could have chosen either payment scheme). If they solve more math puzzles than the other participant, they receive 25 AUD (17 US$) per correctly solved puzzle; if they solve fewer puzzles they receive nothing; in case of a tie they receive 10 AUD (7 US$) per correctly solved puzzle. The choice of payment scheme is our measure of competitiveness (or willingness to compete), where we consider those choosing the tournament scheme as more competitive than those choosing the piece rate scheme.

Patience (time preferences) were measured using a validated survey question from the Global Preferences Survey by Falk et al. (2018). Participants were asked to state using a 7-point Likert scale how willing they are to give up something today to benefit more from that in the future. The question has been validated by showing a positive correlation with incentivized time preference experiments, where individuals choose between a smaller amount that they receive earlier and a larger amount that they receive later (Falk et al. (2018)).

To measure trust and positive reciprocity, we used a version of the Trust Game (TG) (Berg et al. 1995). A first mover (‘Person A’) and a second mover (‘Person B’) are both endowed with 100 AUD (68 US$). Person A can choose to transfer his/her 100 AUD to B. If he/she does not transfer the 100 AUD the game ends. If A transfers his/her 100 AUD, this is tripled and B holds 400 AUD (270 US$). Person B then decides how much of the 400 AUD to return to A. Participants are matched to a random and anonymous other Raine Study participant, and they are equally likely to be in role A or B. Participants make decisions in both roles (A and B) using the strategy method, i.e. before they know in which role they would be, and indicate what they would do in the role of B if A decides to transfer the money. This gives us a binary measure of trust (the decision of A) and a continuous measure between 0 and 400 of positive reciprocity (the decision of B).

We measured negative reciprocity using a version of the Ultimatum Game (UG) (Güth et al. 1982). A first mover (‘Person A’) is endowed with 400 AUD (270 US$) and can propose how to divide the amount (in increments of 50 AUD) between a second mover (‘Person B’) and

him/herself. Person B can then accept or reject the proposed division by A. If B accepts the proposal, the 400 AUD is divided as proposed; if B rejects the proposal both A and B earn nothing. As in the TG, participants make decisions in both roles and we use the strategy method to record decisions. Specifically, participants indicate for each possible proposal by A if they would accept this proposal if they would be in role B. The indicated minimum acceptable offer (MAO) as person B is our measure of negative reciprocity, i.e. a higher MAO indicates stronger negative reciprocity.

Dishonesty was measured using a coin-flipping task (Fischbacher and Föllmi-Heusi 2013). Participants were asked to take a coin and toss it six times. For each time heads comes up they receive 10 AUD, while they receive nothing for each time tails comes up. Participants report the number of heads that came up, which is our measure of dishonesty. Note that this is a probabilistic measure: at the individual level we cannot know whether someone reports truthfully or not.

To provide real financial incentives, 10% of study participants were randomly selected for payment (after the study was closed). Paying a random subsample is frequently used in large-sample studies (e.g. Dohmen et al. 2011). Previous studies suggest that paying a random subsample a (large) amount, does not affect results compared to paying all subjects a (small) amount (e.g. Bolle 1990, Armentier 2006) and there is suggestive evidence that participants prefer the method we use (mentioned in the online appendix of Abdellaoui et al. (2011)).

S2 Experimental instructions and survey questions

Below, we reproduce the experimental instructions, tasks and survey questions that were used in this paper. The full pre-registered experimental and survey design can be found on https://osf.io/xt8s6/?view_only=eb37d6b404e94fd3b9c8952424d588f3

Welcome

Thank you again for participating in this study. Please read the instructions carefully. If you have any questions, please contact us by email.

In total, your participation will last around 35 minutes. If needed, you can always take a break and continue later by logging in again.

Earning money

In this study you can earn money. We will randomly select one out of ten participants who complete the study for payment. The amount of money you earn if you are selected depends on decisions made by you, other participants in this study and chance. If you are selected for payment, a Raine Study representative will contact you to transfer the money to your bank account. Because the amount you earn may depend on the decisions of other participants, your payment is determined after the study period.

What can you expect?

The study consists of 4 parts. In each part, you will make decisions for which you can earn money and we will ask you several questions. At the beginning of each part, you will receive new instructions. In each of the 4 parts you can earn money, but only one of the 4 parts will be randomly selected for payment. This will be done at the end of the study. Thus, because only one part will count, but you don't know which one it will be, it is in your best interest to treat each part as if it is the only one that matters for your final payment.

Payments occur exactly as promised

We are interested in your choices, and we are not interested in "tricking" you in any way. If you see anything out of place or if something doesn't make sense, please let us know by email as it is most likely a bug in the software. Remember that all data will be treated strictly confidentially. None of the other participants in this study will ever find out what you decided.

Ready to get started?

Press the Next button to start with Part 1.

NEXT

Part 1

We now start with Part 1 in which you can earn money.

In this part of the study you will choose between six different options, which are listed below. Each option has two possible outcomes (Outcome A or Outcome B) that are equally likely to occur. Think about the flip of a coin: heads (Outcome A) and tails (Outcome B) are equally likely. You select only one of these options by clicking on the appropriate box.

For example: If you select option 4 and Outcome A occurs, you will be paid $325. If Outcome B occurs, you will be paid $100.

Please select one of these six options for how you would like to be paid.

1		2		3		4		5		6	
A	B	A	B	A	B	A	B	A	B	A	B
$175	$175	$225	$150	$275	$125	$325	$100	$375	$75	$440	$10

CONTINUE

Part 2

We now continue with Part 2 in which you can earn money.

Instructions

In this part you are randomly matched with another participant in this study. One of you will be person A, one of you will be person B. Both of you start with $100.

If you and this part are selected for payment, this will hold for the other participant too. This means that your decisions and the decisions of the other participant will determine your earnings and the earnings of the other participant.

First person A makes a choice, then person B responds.

1) Person A can choose to transfer his/her $100 or not.

   If person A transfers $100, it is TRIPLED and given to person B. So person B then has $400 (3 x $100 plus the original $100) and person A has $0. If Person A does not transfer $100, both person A and person B earn $100.

1) If person A transfers $100, Person B can then choose how much of the money he or she wants to transfer back to person A (between $0 and $400). If Person A does not transfer $100, both person A and person B earn $100, and person B cannot transfer anything back.

We will ask you what you would like to do in both roles. At the end of the study, we will assign you and the person you are matched with to one of the roles. So, it is equally likely that your decision as person A or person B will count.

CONTINUE

Part 2

Test questions

You must answer these two questions correctly to be eligible for payment if this part of the study is selected.

What happens if Person A transfers $100 and Person B transfers back $150?

Person A earns: $____

Person B earns: $____

What happens if Person A does not transfer $100?

Person A earns: $____

Person B earns: $____

BACK TO INSTRUCTIONS VERIFY ANSWERS

(give people the option to continue only after giving the correct answers, or after 3 times an incorrect answer)

Part 2

Your decisions

If you are person A, do you want to transfer your $100 to person B?

0 Yes, transfer the $100 to Person B

0 No, keep the $100 and do not transfer to Person B

If you are person B and person A transfers you $100 how much do you want to transfer back to A? Remember that you will then have $400 (3 x $100 + your initial $100). You can transfer back any amount from $0 to $400.

$______

BACK TO INSTRUCTIONS CONFIRM YOUR CHOICE

Questionnaire 2

We would now like to ask some more questions about you.

In comparison to others, are you a person who is generally willing to give up something today in order to benefit from that in the future?
Please use the scale from 1 to 7 below, where a 1 means you are completely unwilling to give up something today and a 7 means you are very willing to give up something today. You can also use the values in-between to indicate where you fall on the scale.

completely unwilling to give up something today							very willing to give up something today
	1	2	3	4	5	6	7
	O	O	O	O	O	O	O

How do you see yourself: Are you a person who is generally willing to take risks, or do you try to avoid taking risks?
Please use the scale from 1 to 7 below, where a 1 means you are completely unwilling to take risks and a 7 means you are very willing to take risks. You can also use the values in-between to indicate where you fall on the scale.

completely unwilling to take risks							very willing to take risks
	1	2	3	4	5	6	7
	O	O	O	O	O	O	O

Part 3

We now continue with Part 3 in which you can earn money.

Instructions

In this part we ask you to solve math puzzles. Each math puzzle consists of eight numbers, of which two add up to exactly 10. The goal is to select those two numbers. Before this part starts, we offer a practice round. After the practice round, two rounds follow in which you can earn money. In each round you get 3 minutes to solve as many math puzzles as possible.

CONTINUE

Part 3

Instructions

After the following explanation, the practice round will begin. When you click "continue", a box appears on the screen which contains eight numbers between 0 and 10. The goal is to click on those two numbers that add up to exactly 10. When you have clicked the correct two numbers, a new box will appear.

Below you find an example of a box, with the solution next to it:

☐ 6.71	☐ 9.72	☐ 8.87
☐ 5.05		☐ 0.28
☐ 0.26	☐ 9.44	☐ 1.86

☐ 6.71	☑ 9.72	☐ 8.87
☐ 5.05		☑ 0.28
☐ 0.26	☐ 9.44	☐ 1.86

In contrast to the real math puzzle, the practice round has no time limit. When you have solved three math puzzles, you can choose to do another practice round, or to continue with the real task.
Click "continue" to start the practice round.

BACK **CONTINUE**

Part 3

Practice round (3 math puzzles)

Number of correct math puzzles: 0 *[count of correct* math *puzzles]*

Select the two numbers that precisely add up to 10.

☐ 6.71	☐ 9.72	☐ 8.87
☐ 5.05		☐ 0.28
☐ 0.26	☐ 9.44	☐ 1.86

Part 3

Repeat practice round

This was the practice round. Would you like another practice round before the real task begins?
0 Yes, I would like to practice one more time
0 No, I would like to continue to the real task

Part 3

Instructions round 1

After these instructions, the first round will start, in which you can earn money for each correctly solved math puzzle. You get 3 minutes to solve as many math puzzles as possible.

For each math puzzle you solve within these 3 minutes, you receive $10.

When you click "continue" at the bottom of this screen, the 3 minute countdown starts. A counter in the left top of the screen indicates how much time you have left. One by one, math puzzles with numbers will appear. As soon as you have selected those two numbers that exactly add up to 10, a new math puzzle will appear. The top left of the screen shows the time left as well as the number of correctly solved math puzzles.

When the time is up, you can no longer select numbers. Then click "continue" to continue with the study.

As soon as you click "continue", the first math puzzle appears and the timer starts counting down.

CONTINUE

Part 3

Round 1

Time: 3:00 ***[timer counts down from 3 minutes]***
Number of correct math puzzles: 0 *[count of correct math puzzles]*
Select the two numbers that precisely add up to 10.

☐ 6.25	☐ 9.81	☐ 1.46
☐ 6.50		☐ 2.46
☐ 7.10	☐ 4.10	☐ 7.54

Part 3

Instructions round 2

Before the start of the second round, we ask you to choose the way in which you will earn money. There are two payment schemes: ‘piece rate’ and ‘tournament’.

1) If you choose to be paid according to ‘**piece rate**’, you receive $10 for each math puzzle you solved, just as in the previous round.

2) If you choose to be paid according to ‘**tournament’**, your performance will be evaluated relative to the performance of another randomly chosen participant. If your score is higher than this other participant, you receive $25 for each math puzzle you solved. If your score is lower, you receive nothing. In case of a tie, you receive $10 per correct math puzzle.

Which payment scheme do you choose?

0 **Piece rate**, thus $10 per correct math puzzle, regardless of how other participants perform.

0 **Tournament**, thus $25 per correct math puzzle if you do better than another randomly chosen opponent, and $0 if you do worse.

Part 3

Round 2

Time: 3:00 ***[timer counts down from 3 minutes]***
Number of correct math puzzles: 0 *[count of correct math puzzles]*
Select the two numbers that precisely add up to 10.

☐ 6.25	☐ 9.81	☐ 1.46
☐ 6.50		☐ 2.46
☐ 7.10	☐ 4.10	☐ 7.54

Part 3

Other question

If you answer the next question correctly, you will receive an additional $25.
We will compare the number of math puzzles you solved correctly in round 1 to the score of 10 randomly selected other participants in this study.
Out of these 10 participants, how many do you think will have a lower score than you?

0	1	2	3	4	5	6	7	8	9	10
My score will be the lowest										My score will be the highest

Part 4

We now continue with Part 4 in which you can earn money.

Instructions

In this part you are randomly matched with another participant in this study. One of you will be person A, one of you will be person B. Person A starts with $400, person B starts with $0.

If you and this part are selected for payment, this will hold for the other participant too. This

means that your decisions and the decisions of the other participant will determine your earnings and the earnings of the other participant.

First person A makes a choice, then person B responds.

1) Person A can choose to offer any amount between $0 and $400 (in multiples of $50) to person B and keep the rest of the $400 to him- or herself.

2) Person B then chooses to accept or reject the proposal by person A. If Person B accepts the proposal, the $400 will be divided as proposed by person A. If Person B rejects the proposal, both person A and person B earn nothing.

We will ask you what you would like to do in both roles. At the end of the study we will assign you and the person to whom you are matched to one of the two roles. So, it is equally likely that your decision as person A or person B will count.

CONTINUE

Part 4

Test questions

You must answer these two questions correctly to be eligible for payment if this part of the study is selected.

What happens if Person A offers $150 to Person B and Person B **accepts** this proposal?

Person A earns: $____

Person B earns: $____

What happens if Person A offers $150 to Person B and Person B **rejects** this proposal?

Person A earns: $____

Person B earns: $____

BACK TO INSTRUCTIONS VERIFY ANSWERS

(give people the option to continue only after giving the correct answers, or after 3 times an incorrect answer)

Part 4

Your decisions

If you are person B, which proposals by person A would you accept and which ones would you reject?

Amount offered to person B	$0	$50	$100	$150	$200	$250	$300	$350	$400
	accept	accept	accept	accept	accept	accept	accept	accept	accept
	reject	reject	reject	reject	reject	reject	reject	reject	reject

BACK TO INSTRUCTIONS CONFIRM YOUR CHOICE

Part 4

Your decisions

If you are person A, how much do you want to offer to person B? (select one proposal)

Amount offered to person B	$0	$50	$100	$150	$200	$250	$300	$350	$400

BACK TO INSTRUCTIONS CONFIRM YOUR CHOICE

Final task

Finally we would like you to do the following. Please take a coin and flip it 6 times. For each time that the outcome is heads you will receive an additional $10 if you are selected for payment. For each time that the outcome is tails, you will receive nothing.

Out of your 6 coin tosses, how often did heads come up?

_______ times heads

S3 Additional analyses

Sex differences

The left panel in Fig. 2 in the main text shows the estimated sex differences. For comparison, all non-binary outcome measures (both measures of risk tolerance, patience, positive and negative reciprocity, dishonesty) have been standardized to have mean zero and a standard deviation equal to one. All estimates underlying the left panel in Fig. 2 can be found in the respective column (2) of Table S3. Fig. S2 provides distributions of each economic preference measure by sex.

The observed sex differences in risk attitudes are consistent with a large literature documenting that men are more risk tolerant than women (cf. Eckel and Grossman 2002; Croson and Gneezy 2009; Dohmen et al. 2011; Charness and Gneezy 2012; Niederle 2016; Falk et al. 2018; but see also Nelson 2015; Filippin and Crosetto 2016; and in turn Charness et al. 2018). We find that, using the incentivized measure, men are 42% of a standard deviation more risk tolerant than women (b = 0.420, $t(740)$ = 5.72, 95% CI = [0.276, 0.565], n = 742, $p < 0.001$) and 46% of a standard deviation more risk tolerant when using the self-reported measure (b = 0.458, $t(692)$ = 6.07, 95% CI = [0.310, 0.606], n = 694, $p < 0.001$).

We observe that men are more competitive than women: 45% of men choose the competitive tournament payment scheme compared to 25% of women (b = 0.198, $t(676)$ = 5.50, 95% CI = [0.128, 0.269], n = 678, $p < 0.001$). This is in line with previous findings using similar tasks (Niederle and Vesterlund 2011; Niederle 2016).

For time preferences, the observation that men are more patient than women is consistent with previous findings by Falk et al. (2018), who use the same measure of time preferences as we do. Compared to the literature on sex differences and risk attitudes or competitiveness, there are much fewer studies on sex differences in time preferences and hence, the evidence is less clear-cut. For example, Sutter et al. (2013) and Almås et al. (2015) find no clear sex difference in time preferences, and Meier and Sprenger (2013) find that women are more patient than men. We find that self-reported patience of men is 18% of a standard deviation higher than that of women (b = 0.179, $t(692)$ = 2.32, 95% CI = [0.028, 0.330], n = 694, p = 0.021).

For social preferences, the sex differences are less pronounced. While we find sex differences in negative reciprocity, we find no differences in trust, positive reciprocity and dishonesty. This is

in line with previous studies that find that sex differences in social preferences are not very robust (Croson and Gneezy 2009; Balliet et al. 2011; Niederle 2016). In the TG, men choose to transfer (‘trust’) in 76% of the cases, while women transfer (‘trust’) in 71% of the cases (b = 0.047, t(693) = 1.37, 95% CI = [-0.020, 0.114], n = 695, p = 0.171). Also for second movers in the TG (our measure of positive reciprocity), we observe no sex difference (b = -0.025, t(692) = -0.32, 95% CI = [-0.177, 0.127], n = 694, p = 0.750). For negative reciprocity, we do observe strong sex differences in the UG. Men are more likely to reject low offers: they have a minimum acceptable offer (MAO) that is 27% of a standard deviation larger than women (b = 0.270, t(661) = 3.45, 95% CI = [0.116, 0.424], n = 663, p = 0.001). For dishonesty, we observe that men report somewhat more successful coin tosses than women, which suggests they are lying slightly more than women, on average. We find a difference of 14% of a standard deviation, which is not statistically significant at the 5% level (b = 0.139, t(659) = 1.76, 95% CI = [-0.016, 0.294], n = 661, p = 0.078). In a recent meta-analysis, Abeler et al. (2019) find that women tend to behave less dishonestly on average.

Robustness

Some previous papers investigated non-linear effects of 2D:4D (Brañas-Garza et al. 2013; Galizzi and Nieboer 2015; Parslow et al. 2019), or used the average 2D:4D from both hands (Sapienza et al. 2009). Neither taking average 2D:4D nor including a squared term affect our results (see Fig. S5). Only for the incentivized risk tolerance measure we find a significant positive (but small) coefficient on the squared term. While this suggests that those with particularly high or low 2D:4D are more likely to take risks, this finding should be interpreted with caution as it would not have survived even a minimal correction for two hypotheses. We also tested possible non-linear associations between testosterone at birth and economic preferences. Fig. S6 shows the effects of taking the natural logarithm of total testosterone at birth, as well as including squared terms. Again, we find results similar to when we assume a linear effect.

We also consider only those participants for whom we are confident that they fully understood the experimental tasks. In the instructions for the TG and UG, we included a range of test questions to check if people understood the instructions. Ninety-seven participants did not

answer all test questions correctly (out of 663 who answered these questions). Fig. S7 shows the associations between sex, testosterone at birth and 2D:4D, but now only for those participants who did answer all test questions correctly. The results are virtually identical to when we include all participants (see Fig. 2 in the main text).

Previous studies have suggested that sex differences in 2D:4D may not be universal (see Apicella et al. 2016 and the references therein) and several studies find differential associations between 2D:4D and economic preferences depending on ethnicity (see Brañas-Garza et al. 2018 and the references therein). Our sample consists largely of Caucasian participants: for 87 percent of participants, both parents are reported to be Caucasian (see also Table S2). If we focus on this subsample of participants with two Caucasian parents, none of the associations between testosterone at birth or 2D:4D and economic preferences is statistically significant. Fig. S8 summarizes the results for this subsample.

Fig. S9 shows associations between estrogen measures (estradiol (E2) and BioE2) and economic preferences. All associations are nonsignificant.

S4 Figures and Tables

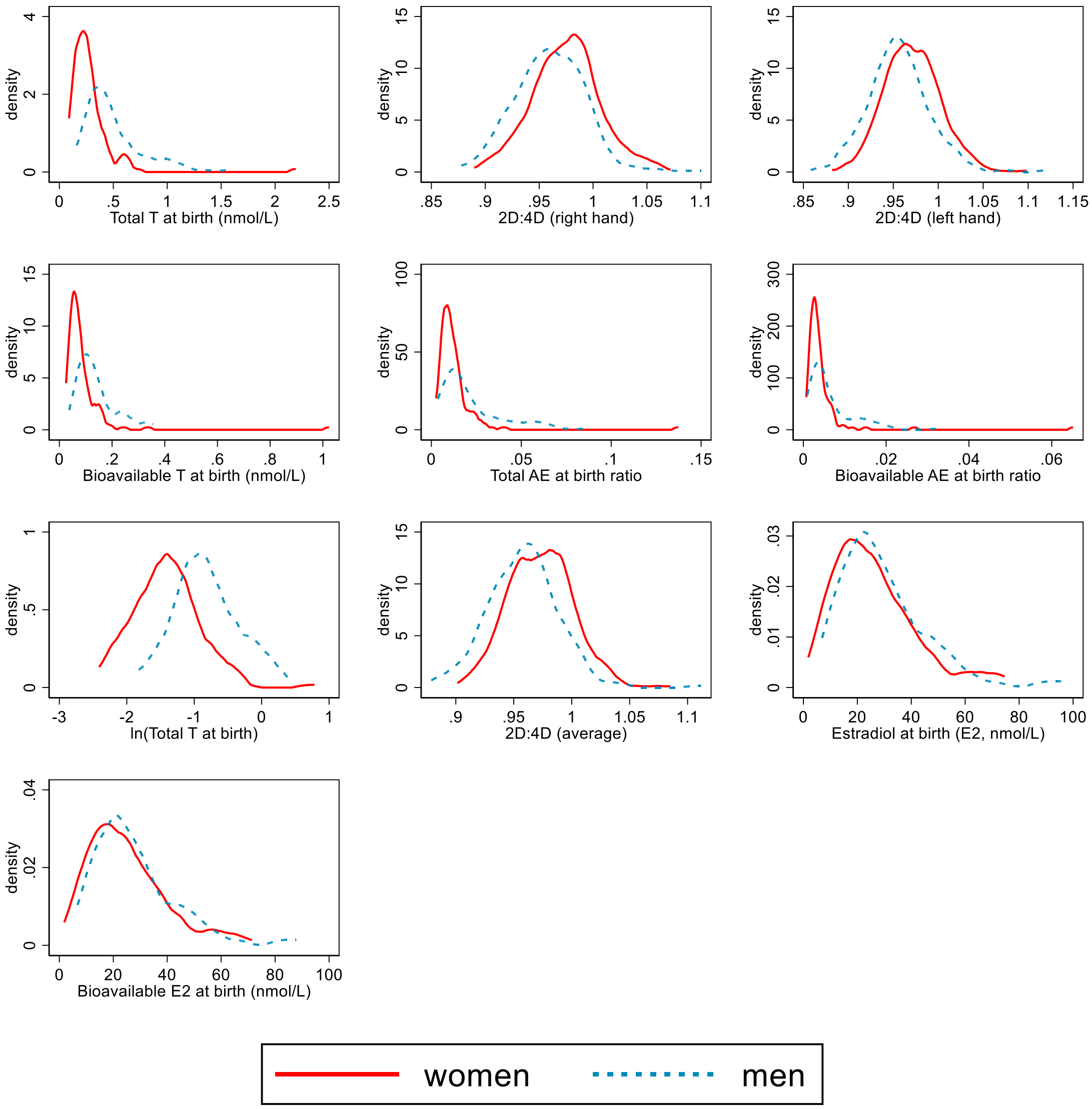

Fig. S1. Kernel density plots of hormone measures at birth and 2D:4D, by sex. Plots based on participants who participated in our study. For Total T at birth, 2D:4D (right hand), 2D:4D (left hand), Bioavailable T at birth, Total AE at birth ratio, Bioavailable AE at birth, ln(Total T at birth), 2D:4D (average), we observe significant sex differences in the distributions (Kolmogorov-Smirnov tests, all $p < 0.001$). For Estradiol (E2) at birth and Bioavailable E2 at birth, the sex differences are not statistically significant (Kolmogorov-Smirnov tests, all $p = 0.210$ for Estradiol (E2), $p = 0.253$) Sample sizes: Total T at birth: 125 women and 92 men; 2D:4D (right hand): 350 women and 247 men; 2D:4D (left hand): 349 women and 246 men; ln(Total T at birth): 125 women and 92 men; Bioavailable T at birth: 124 women and 92 men; Total AE at birth ratio: 125 women and 92 men; Bioavailable AE at birth ratio: 124 women and 92 men; 2D:4D (average): 349 women and 246 men; Estradiol (E2) at birth: 132 women and 93 men; Bioavailable E2 at birth: 131 women and 93 men.

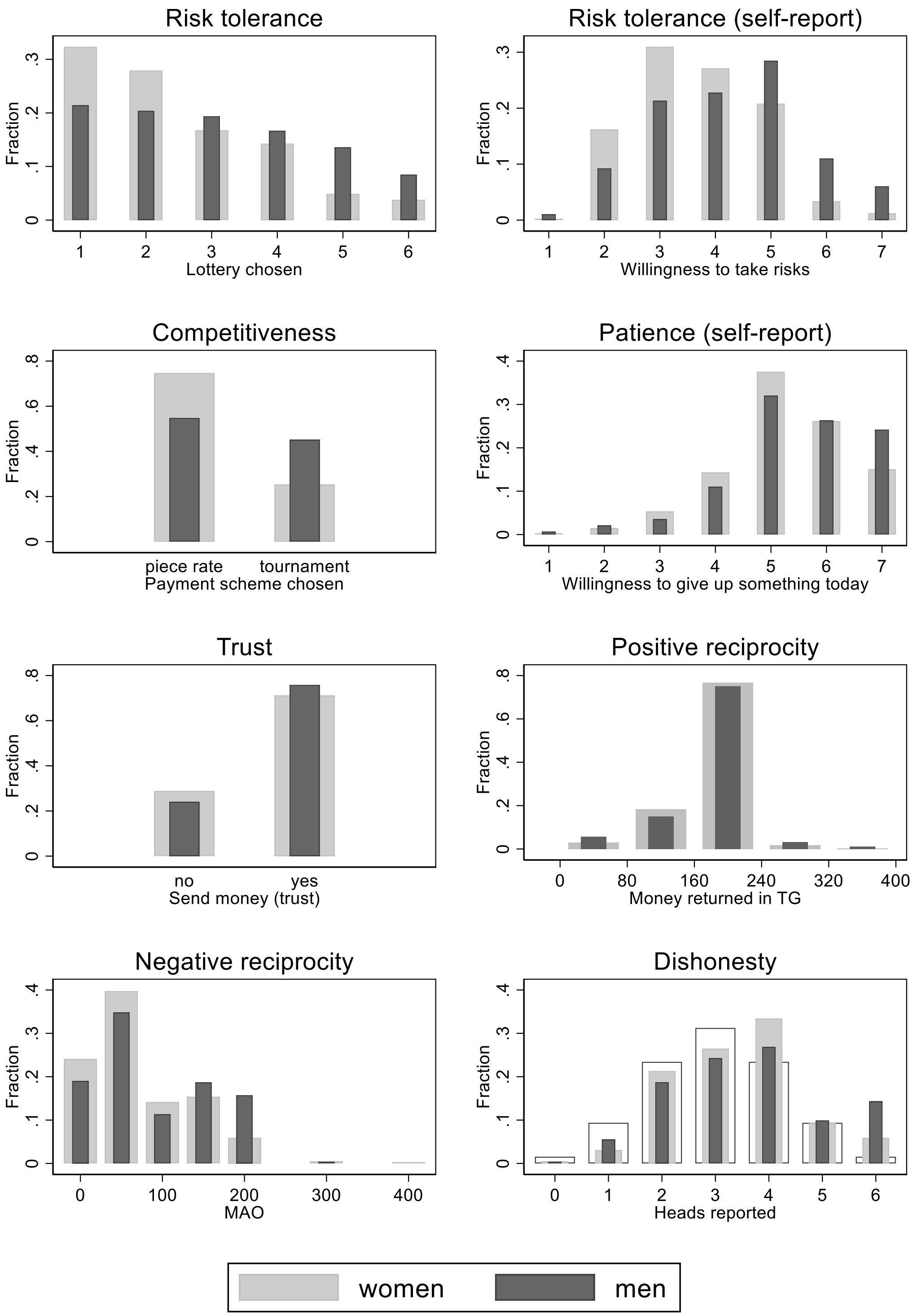

Fig. S2. Sex differences in economic preferences measures. Distributions of economic preference measures, by sex. For positive reciprocity, all amounts returned are grouped in bins of 80 AUD (e.g. 0-79 is the first bin, 80 to 159 is the second bin, etc.). For dishonesty, the white bars indicate the expected distribution if all subjects would report truthfully.

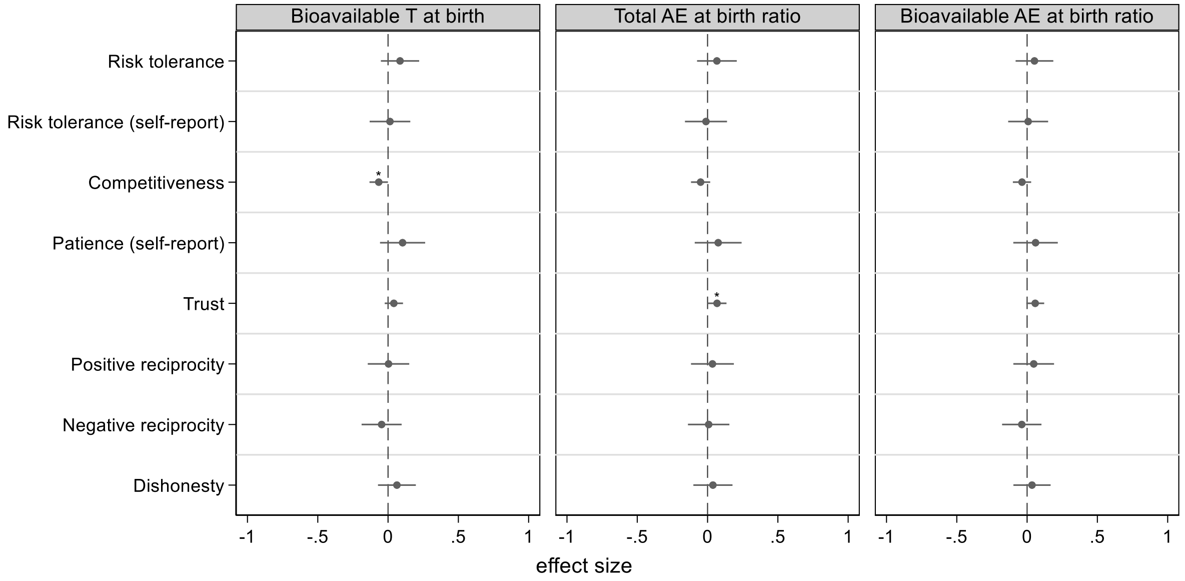

Fig. S3. Other (pre-registered) neonatal hormone measures and economic preferences. Point estimates and 95% confidence intervals from OLS regressions where the respective dependent variable is regressed on the respective hormone measure and a sex dummy. * $p < 0.05$, ** $p < 0.01$, *** $p < 0.005$. Competitiveness and Trust are binary measures, all other measures (incl. T and AE measures) are standardized to have mean 0 and a standard deviation of 1.

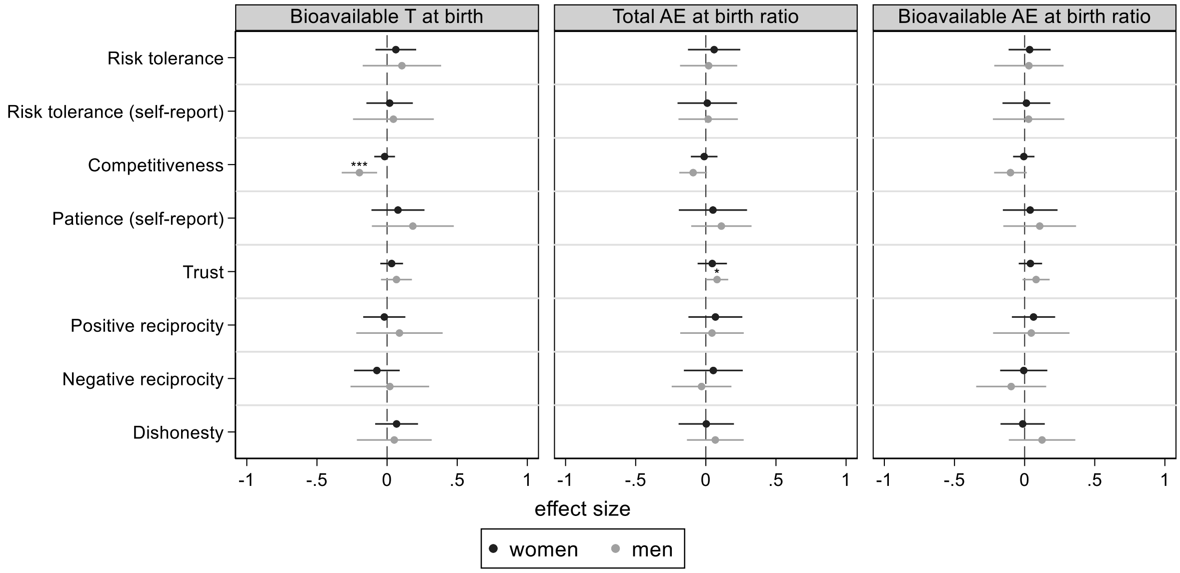

Fig. S4. Other (pre-registered) neonatal hormone measures and economic preferences (by sex). Point estimates and 95% confidence intervals from OLS regressions where the respective dependent variable is regressed on the respective hormone measure, separately for women and men. * $p < 0.05$, ** $p < 0.01$, *** $p < 0.005$. Competitiveness and Trust are binary measures, all other measures (incl. T and AE measures) are standardized to have mean 0 and a standard deviation of 1.

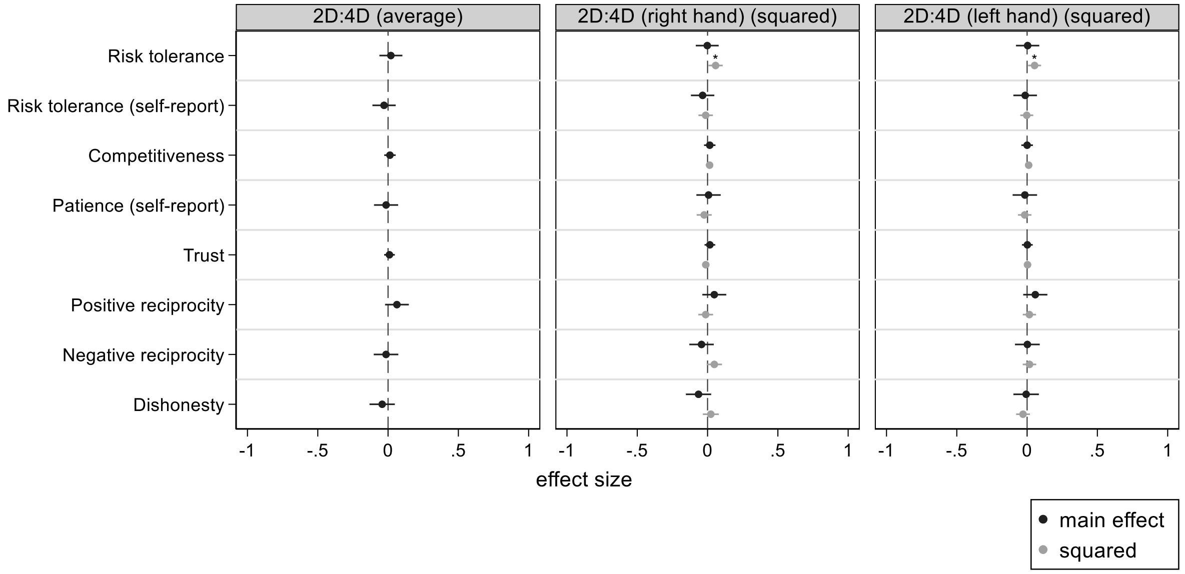

Fig. S5. Average 2D:4D and nonlinear effects of 2D:4D. Point estimates and 95% confidence intervals from OLS regressions where the respective dependent variable is regressed on the average 2D:4D of both hands and a sex dummy (left panel); second order polynomials of 2D:4D and a sex dummy (middle and right panel). * $p < 0.05$, ** $p < 0.01$, *** $p < 0.005$. Sex, Competitiveness and Trust are binary measures, all other measures (incl. 2D:4D) are standardized to have mean 0 and a standard deviation of 1.

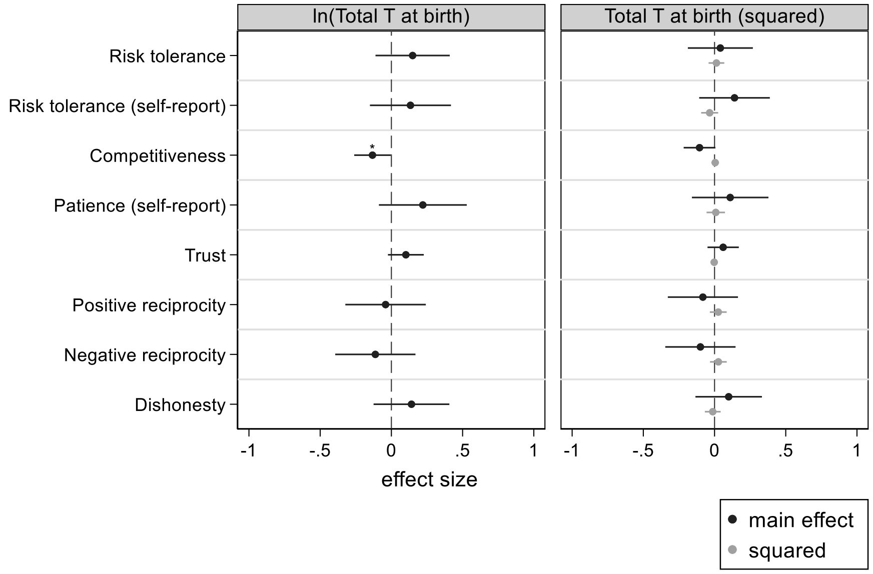

Fig. S6. Nonlinear effects of T at birth and economic preferences. Point estimates and 95% confidence intervals from OLS regressions where the respective dependent variable is regressed on ln(total T at birth) and a sex dummy (left panel); a second order polynomial of Total T at birth (standardized to have mean 0 and a standard deviation of 1) and a sex dummy (right panel). * $p < 0.05$, ** $p < 0.01$, *** $p < 0.005$. Sex, Competitiveness and Trust are binary measures, all other dependent variables and total T at birth are standardized to have mean 0 and a standard deviation of 1. ln(Total T at birth) has not been standardized.

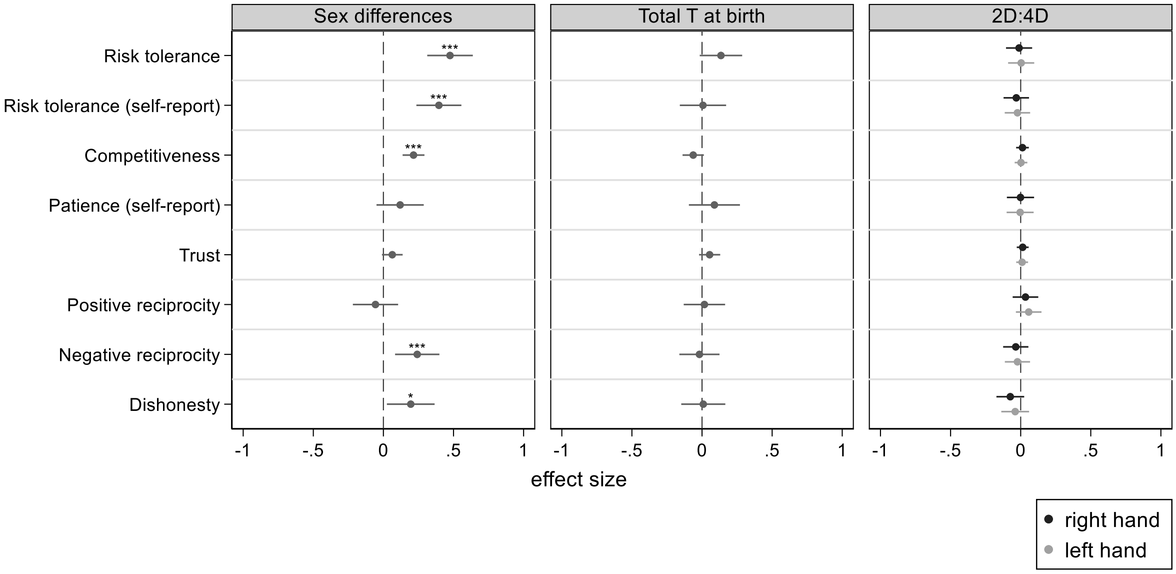

Fig. S7. Sex differences, T at birth, 2D:4D and economic preferences – Only for those participants that answered all test questions correctly. Point estimates and 95% confidence intervals from OLS regressions where the respective dependent variable is regressed on a dummy that equals 1 for men and 0 for women (left panel), on T at birth and a sex dummy (middle panel) or the left or right hand 2D:4D ratio and a sex dummy (right panel). Based only on those participants that answered all test questions (for understanding of the UG and TG) correctly. * $p < 0.05$, ** $p < 0.01$, *** $p < 0.005$. Sex, Competitiveness and Trust are binary measures, all other measures (incl. T and 2D:4D) are standardized to have mean 0 and a standard deviation of 1.

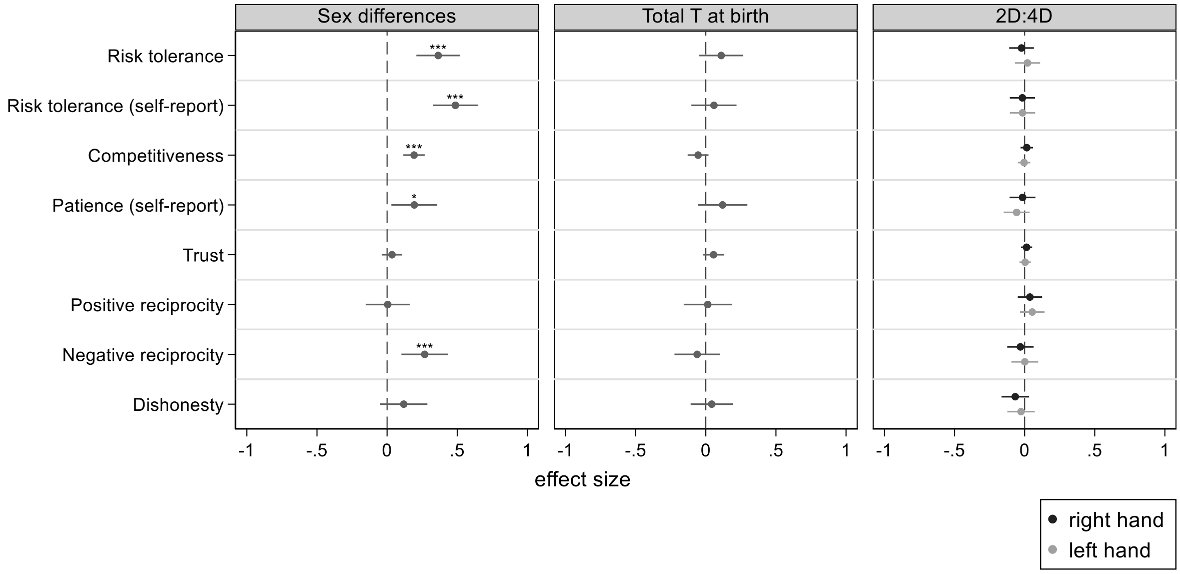

Fig. S8. Sex differences, T at birth, 2D:4D and economic preferences – Only for participants with two Caucasian parents. Point estimates and 95% confidence intervals from OLS regressions where the respective dependent variable is regressed on a dummy that equals 1 for men and 0 for women (left panel), on T at birth and a sex dummy (middle panel) or the left or right hand 2D:4D ratio and a sex dummy (right panel). Based only on those participants for whom both parents reported to be Caucasian. $^{*}p < 0.05$, $^{**}p < 0.01$, $^{***}p < 0.005$. Sex, Competitiveness and Trust are binary measures, all other measures (incl. T and 2D:4D) are standardized to have mean 0 and a standard deviation of 1.

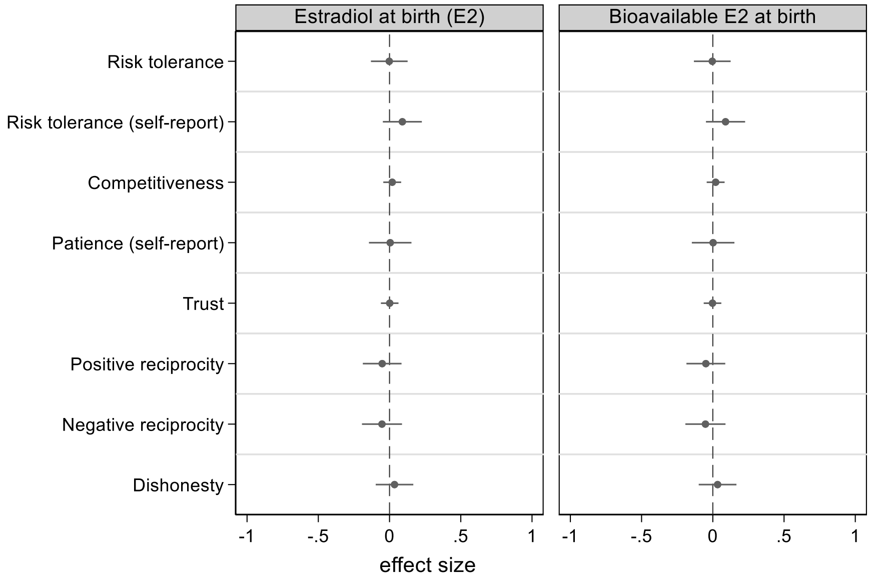

Fig. S9. Estradiol (E2), BioE2 at birth and economic preferences. Point estimates and 95% confidence intervals from OLS regressions where the respective dependent variable is regressed on the respective hormone measure and a sex dummy. * $p < 0.05$, ** $p < 0.01$, *** $p < 0.005$. Competitiveness and Trust are binary measures, all other measures (incl. the E2 and BioE2 measures) are standardized to have mean 0 and a standard deviation of 1.

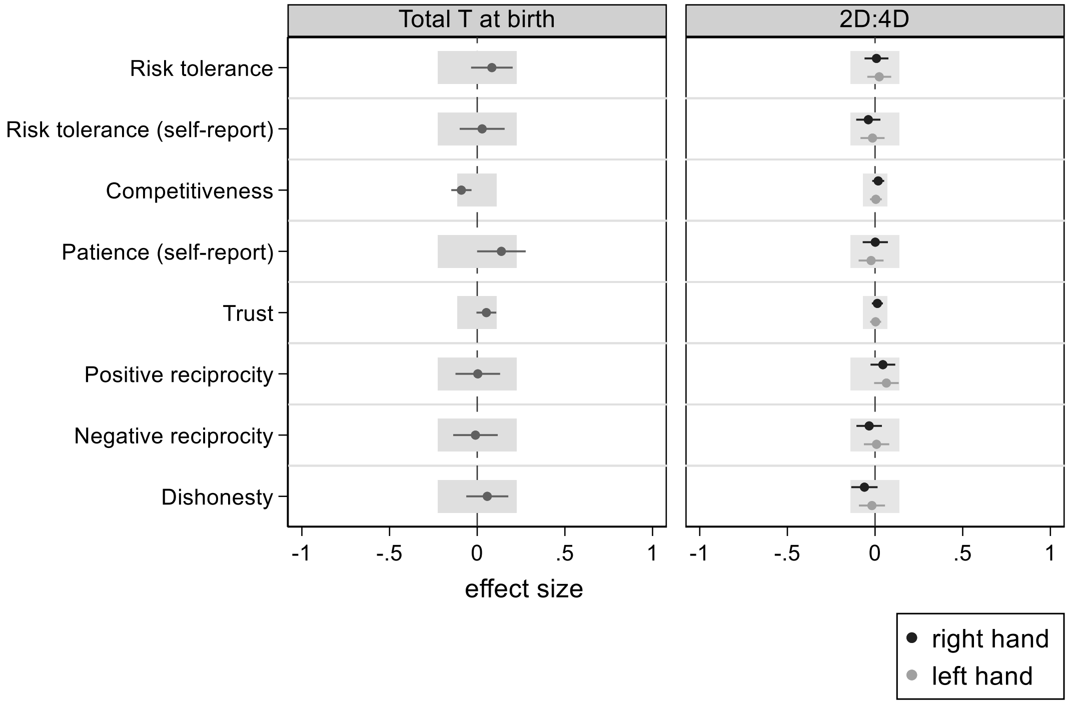

Fig. S10. Equivalence tests for T at birth, 2D:4D and economic preferences. Point estimates and 90% confidence intervals from OLS regressions where the respective dependent variable is T at birth and a sex dummy (left panel) or the left or right hand 2D:4D ratio and a sex dummy (right panel). Shaded areas indicate effect sizes smaller than the smallest effect size we have sufficient power to detect ($\alpha = 0.05$ and power = 0.90). Sex, Competitiveness and Trust are binary measures, all other measures (incl. T and 2D:4D) are standardized to have mean 0 and a standard deviation of 1.

	Total T at birth (nmol/L)	ln(Total T at birth)	BioT at birth (nmol/L)	Total AE at birth ratio	BioAE at birth ratio	2D:4D (right hand)	2D:4D (left hand)	2D:4D (average)	Estradiol at birth (E2, nmol/L)	Bioavailable E2 at birth (nmol/L)
Total T at birth (nmol/L)	-	0.954*** (92)	0.923*** (92)	0.629*** (92)	0.549*** (92)	-0.00326 (78)	0.0602 (77)	0.0299 (77)	0.0304 (92)	0.0214 (92)
ln(Total T at birth)	0.844*** (125)	-	0.909*** (92)	0.639*** (92)	0.577*** (92)	-0.0578 (78)	0.0199 (77)	-0.0200 (77)	0.0298 (92)	0.0229 (92)
Bioavailable T at birth (nmol/L)	0.952*** (124)	0.699*** (124)	-	0.659*** (92)	0.670*** (92)	-0.0324 (78)	0.00356 (77)	-0.0180 (77)	-0.0347 (92)	-0.0302 (92)
Total AE at birth ratio	0.787*** (125)	0.474*** (125)	0.865*** (124)	-	0.964*** (92)	0.117 (78)	0.0894 (77)	0.118 (77)	-0.558*** (92)	-0.562*** (92)
Bioavailable AE at birth ratio	0.766*** (124)	0.429*** (124)	0.884*** (124)	0.971*** (124)	-	0.0931 (78)	0.0659 (77)	0.0915 (77)	-0.549*** (92)	-0.547*** (92)
2D:4D (right hand)	-0.0681 (90)	-0.0734 (90)	-0.0707 (89)	-0.104 (90)	-0.0802 (89)	-	0.651*** (246)	0.902*** (246)	-0.112 (79)	-0.107 (79)
2D:4D (left hand)	-0.187 (90)	-0.235* (90)	-0.188 (89)	-0.133 (90)	-0.129 (89)	0.573*** (349)	-	0.915*** (246)	-0.0430 (78)	-0.0457 (78)
2D:4D (average)	-0.141 (90)	-0.169 (90)	-0.143 (89)	-0.132 (90)	-0.116 (89)	0.889*** (349)	0.885*** (349)	-	-0.0945 (78)	-0.0935 (78)
Estradiol at birth (E2, nmol/L)	0.217* (125)	0.421*** (125)	0.0739 (124)	-0.323*** (125)	-0.266*** (124)	-0.0291 (96)	-0.113 (96)	-0.0789 (96)	-	0.999*** (93)
Bioavailable E2 at birth (nmol/L)	0.221* (124)	0.425*** (124)	0.0854 (124)	-0.316*** (124)	-0.261*** (124)	-0.0435 (95)	-0.124 (95)	-0.0934 (95)	0.998*** (131)	-

Table S1. Correlations between hormone measures and digit ratios. Correlation coefficients, number of observations in parentheses. Entries below the diagonal indicate correlations for women, entries above the diagonal indicate correlations for men. * $p < 0.05$, ** $p < 0.01$, *** $p < 0.005$.

	N	Mean	St. dev.	Min	Max
**Demographic**					
Female	742	.604	.489	0	1
Age (years)	742	26.5	.709	25.3	27.8
**Hormones and digit ratios**					
Total T at birth (nmol/L)	217	.374	.263	.0898	2.19
Total T at birth (ln)	217	-1.16	.577	-2.41	.785
Bioavailable T at birth (nmol/L)	216	.109	.0907	.0256	1.02
Total AE at birth ratio	217	.0167	.0157	.00267	.137
Bioavailable AE at birth ratio	216	.00525	.00609	.00071	.0652
2D:4D (right hand)	597	.969	.0329	.878	1.1
2D:4D (left hand)	595	.965	.0334	.858	1.12
2D:4D (average)	595	.967	.0298	.879	1.11
Estradiol at birth (E2, nmol/L)	225	27.7	15.9	1.84	95.6
Bioavailable E2 at birth (nmol/L)	224	26.2	14.8	1.77	87.9
**Economic preferences measures**					
Risk tolerance	742	2.68	1.51	1	6
Risk tolerance (self-report)	694	3.91	1.28	1	7
Competitiveness	678	.335	.472	0	1
Patience (self-report)	694	5.35	1.2	1	7
Trust	695	.731	.444	0	1
Money returned in TG (positive reciprocity)	694	183	50.1	0	400
MAO (negative reciprocity)	663	78.8	66.6	0	400
Heads reported (dishonesty)	661	3.48	1.31	0	6
**Controls for competitiveness measure**					
Puzzles solved (part 1)	678	9.45	4.44	0	25
Confidence (math task)	676	5.18	2.15	0	10
**Fetal growth controls**					
Gestation time (days)	741	275	14.9	172	299
Birth weight (grams)	742	3319	586	785	5110
**Neonatal SES controls**					
Family income (yearly) (N = 705)	N	%			
Less than $7,000	42	6.0			
$7,000 - $11,999	39	5.5			
$12,000 - $23,999	147	20.9			
$24,000 - $35,000	202	28.7			
$36,000 or more	275	39.0			
Mother’s degree (N = 728)	N	%			
None	309	42.4			
Trade certificate or apprenticeship	63	8.7			
Professional registration (non-degree)	85	11.7			
College Diploma or Degree	125	17.2			
University degree	106	14.6			
Other	40	5.5			

Table S2: Descriptive statistics (continues on next page)

Father’s degree (N = 642)	N	%
None	161	25.1
Trade certificate or apprenticeship	190	29.6
Professional registration (non-degree)	29	4.5
College Diploma or Degree	87	13.6
University degree	151	23.5
Other	24	3.7

	N	Mean	St. dev.	Min	Max
Family size	723	2.53	.752	1	5
Mother's years of schooling	727	11.1	1.02	8	12
Father's years of schooling	638	11	1.18	2	12
Mother's age at birth	728	29.4	5.54	15.3	46.6
Father's age at birth	722	31.5	6.52	15	58
Parents live together	723	.927	.261	0	1
Mother born in Australia	728	.574	.495	0	1
Father born in Australia	728	.538	.499	0	1

Parent’s race

Mother’s race (N = 728)	N	%
Caucasian (European descent)	662	90.9
Aboriginal	2	0.3
Polynesian	5	0.7
Vietnamese	3	0.4
Chinese	34	4.7
Indian	16	2.2
Other	6	0.8

Father’s race (N = 728)	N	%
Caucasian (European descent)	656	90.1
Aboriginal	2	0.3
Polynesian	6	0.8
Vietnamese	2	0.3
Chinese	27	3.7
Indian	19	2.6
Other	10	1.4
Unknown	6	0.8

Table S2: Descriptive statistics (continued)

	(1)	(2)	(3)	(4)	(5)	(6)	(7)	(8)	(9)	(10)	(11)	(12)	(13)	(14)	(15)	(16)	(17)	(18)	(19)	(20)	(21)	(22)	(23)	(24)	(25)	(26)
Sample:	All available data																Test Q correct	Caucasian	Women				Men			
Total T at birth	0.159*		0.084	0.108	0.114	0.102								0.041			0.135	0.110	0.108				0.063			
	(0.066)		(0.072)	(0.106)	(0.085)	(0.120)								(0.116)			(0.077)	(0.079)	(0.095)				(0.111)			
Male		0.420***	0.369*	0.371*	0.397*	0.396*	0.388**	0.381**	0.417***	0.417***	0.418***	0.419***	0.350*	0.398*	0.409***	0.402***	0.458***	0.343*								
		(0.074)	(0.145)	(0.145)	(0.169)	(0.170)	(0.138)	(0.140)	(0.139)	(0.139)	(0.135)	(0.136)	(0.154)	(0.158)	(0.132)	(0.132)	(0.156)	(0.158)								
Male X Total T at birth				-0.045		0.022																				
				(0.144)		(0.165)																				
Bioavailable T at birth							0.076	0.062												0.062				0.106		
							(0.068)	(0.082)												(0.073)				(0.141)		
Male X Bioavailable T at birth								0.043																		
								(0.149)																		
Total AE at birth ratio									0.037	0.059											0.059				0.020	
									(0.069)	(0.105)											(0.094)				(0.103)	
Male X Total AE at birth ratio										-0.039																
										(0.139)																
Bioavailable AE at birth ratio											0.034	0.036										0.036				0.031
											(0.067)	(0.085)										(0.076)				(0.124)
Male X Bioavailable AE at birth ratio												-0.005														
												(0.139)														
ln(Total T at birth)													0.149													
													(0.132)													
(Total T at birth)^2														0.013												
														(0.028)												
Estradiol at birth (E2)															-0.002											
															(0.065)											
Bioavailable E2 at birth																-0.004										
																(0.065)										
Fetal growth controls	No	No	No	No	Yes	Yes	No	No	No	No	No	No	No	No	No	No	No	No	No	No	No	No	No	No	No	No
Neonatal SES controls	No	No	No	No	Yes	Yes	No	No	No	No	No	No	No	No	No	No	No	No	No	No	No	No	No	No	No	No
Constant	0.032	-0.166***	-0.124	-0.115	-3.426	-3.487	-0.128	-0.131	-0.144	-0.139	-0.140	-0.140	0.057	-0.150	-0.124	-0.117	-0.166	-0.128	-0.115	-0.131	-0.139	-0.140	0.255*	0.251*	0.278*	0.279*
	(0.066)	(0.046)	(0.090)	(0.094)	(2.127)	(2.181)	(0.088)	(0.089)	(0.088)	(0.090)	(0.087)	(0.088)	(0.206)	(0.105)	(0.085)	(0.085)	(0.096)	(0.096)	(0.084)	(0.079)	(0.081)	(0.079)	(0.125)	(0.122)	(0.119)	(0.117)
Observations	217	742	217	217	184	184	216	216	217	217	216	216	217	217	225	224	171	180	125	124	125	124	92	92	92	92
R^2	0.026	0.042	0.055	0.055	0.193	0.194	0.053	0.053	0.050	0.050	0.048	0.048	0.054	0.056	0.042	0.040	0.102	0.055	0.010	0.006	0.003	0.002	0.004	0.006	0.000	0.001

Table S3a. Hormones and economic preferences, risk tolerance (incentivized measure). OLS regressions, standard errors in parentheses. * $p < 0.05$, ** $p < 0.01$, *** $p < 0.005$. Dep. variable: risk tolerance (lottery chosen). Risk tolerance and all hormone measures except ln(Total T at birth) are standardized to have mean 0 and a standard deviation of 1. Fetal growth controls include birth weight and gestational time. Neonatal SES controls were all recorded during pregnancy and are listed in Table S2. Column (17) is based only on those participants that answered all test questions (for understanding of the UG and TG) correctly. Column (18) is based only on those participants for whom both parents report to be Caucasian.

	(1)	(2)	(3)	(4)	(5)	(6)	(7)	(8)	(9)	(10)	(11)	(12)	(13)	(14)	(15)	(16)	(17)	(18)	(19)	(20)	(21)	(22)	(23)	(24)	(25)	(26)
Sample:	All available data																Test Q correct	Caucasian	Women				Men			
Total T at birth	0.127<br>(0.072)		0.028<br>(0.078)	0.058<br>(0.115)	0.073<br>(0.092)	0.147<br>(0.129)								0.140<br>(0.126)			0.007<br>(0.084)	0.058<br>(0.082)	0.058<br>(0.108)				0.003<br>(0.114)			
Male		0.458***<br>(0.075)	0.495***<br>(0.158)	0.497***<br>(0.159)	0.556***<br>(0.186)	0.573***<br>(0.188)	0.499***<br>(0.151)	0.495***<br>(0.154)	0.510***<br>(0.151)	0.510***<br>(0.151)	0.508***<br>(0.148)	0.506***<br>(0.148)	0.439**<br>(0.168)	0.419*<br>(0.172)	0.496***<br>(0.142)	0.494***<br>(0.142)	0.516***<br>(0.171)	0.463**<br>(0.164)								
Male X Total T at birth				-0.055<br>(0.156)		-0.147<br>(0.181)																				
Bioavailable T at birth							0.026<br>(0.074)	0.018<br>(0.089)												0.018<br>(0.084)				0.045<br>(0.145)		
Male X Bioavailable T at birth								0.027<br>(0.162)																		
Total AE at birth ratio									0.014<br>(0.075)	0.010<br>(0.114)											0.010<br>(0.107)				0.017<br>(0.107)	
Male X Total AE at birth ratio										0.006<br>(0.151)																
Bioavailable AE at birth ratio											0.019<br>(0.072)	0.013<br>(0.091)										0.013<br>(0.086)				0.028<br>(0.128)
Male X Bioavailable AE at birth ratio												0.016<br>(0.151)														
ln(Total T at birth)													0.134<br>(0.144)													
(Total T at birth)^2														-0.034<br>(0.030)												
Estradiol at birth (E2)															0.090<br>(0.069)											
Bioavailable E2 at birth																0.089<br>(0.070)										
Fetal growth controls	No	No	No	No	Yes	Yes	No	No	No	No	No	No	No	No	No	No	No	No	No	No	No	No	No	No	No	No
Neonatal SES controls	No	No	No	No	Yes	Yes	No	No	No	No	No	No	No	No	No	No	No	No	No	No	No	No	No	No	No	No
Constant	-0.051<br>(0.073)	-0.185***<br>(0.048)	-0.263**<br>(0.099)	-0.253*<br>(0.103)	0.081<br>(2.469)	0.578<br>(2.547)	-0.263**<br>(0.097)	-0.265**<br>(0.098)	-0.269**<br>(0.097)	-0.270**<br>(0.099)	-0.266**<br>(0.096)	-0.267**<br>(0.097)	-0.085<br>(0.223)	-0.198<br>(0.114)	-0.273***<br>(0.092)	-0.270***<br>(0.093)	-0.186<br>(0.105)	-0.259*<br>(0.101)	-0.253*<br>(0.097)	-0.265**<br>(0.093)	-0.270***<br>(0.093)	-0.267***<br>(0.091)	0.244<br>(0.130)	0.230<br>(0.127)	0.240<br>(0.122)	0.240*<br>(0.120)
Observations	208	694	208	208	176	176	207	207	208	208	207	207	208	208	215	214	171	173	118	117	118	117	90	90	90	90
R^2	0.015	0.051	0.059	0.060	0.211	0.215	0.059	0.059	0.059	0.059	0.058	0.058	0.063	0.065	0.066	0.065	0.063	0.063	0.002	0.000	0.000	0.000	0.000	0.001	0.000	0.001

Table S3b. Hormones and economic preferences, risk tolerance (self-reported measure). OLS regressions, standard errors in parentheses. * $p < 0.05$, ** $p < 0.01$, *** $p < 0.005$. Dep. variable: risk tolerance (self-reported). Risk tolerance and all hormone measures except ln(Total T at birth) are standardized to have mean 0 and a standard deviation of 1. Fetal growth controls include birth weight and gestational time. Neonatal SES controls were all recorded during pregnancy and are listed in Table S2. Column (17) is based only on those participants that answered all test questions (for understanding of the UG and TG) correctly. Column (18) is based only on those participants for whom both parents report to be Caucasian.

	(1)	(2)	(3)	(4)	(5)	(6)	(7)	(8)	(9)	(10)	(11)	(12)	(13)	(14)
Sample:	All available data													
Total T at birth	-0.034<br>(0.033)		-0.090*<br>(0.035)	-0.007<br>(0.050)	-0.010<br>(0.047)	-0.099*<br>(0.041)	-0.033<br>(0.057)	-0.042<br>(0.055)						
Male		0.198***<br>(0.036)	0.283***<br>(0.070)	0.291***<br>(0.070)	0.207***<br>(0.068)	0.295***<br>(0.082)	0.310***<br>(0.082)	0.212*<br>(0.081)	0.247***<br>(0.068)	0.277***<br>(0.068)	0.240***<br>(0.068)	0.240***<br>(0.068)	0.222***<br>(0.067)	0.229***<br>(0.067)
Male X Total T at birth				-0.159*<br>(0.069)	-0.167*<br>(0.065)		-0.129<br>(0.079)	-0.136<br>(0.076)						
Bioavailable T at birth									-0.070*<br>(0.033)	-0.017<br>(0.039)				
Male X Bioavailable T at birth										-0.180*<br>(0.072)				
Total AE at birth ratio											-0.055<br>(0.034)	-0.012<br>(0.051)		
Male X Total AE at birth ratio												-0.079<br>(0.069)		
Bioavailable AE at birth ratio													-0.040<br>(0.033)	-0.006<br>(0.041)
Male X Bioavailable AE at birth ratio														-0.095<br>(0.068)
ln(Total T at birth)														
(Total T at birth)^2														
Estradiol at birth (E2)														
Bioavailable E2 at birth														
Risk tolerance					0.053<br>(0.032)			0.073<br>(0.038)						
Puzzles solved (part 1)					-0.004<br>(0.008)			0.001<br>(0.010)						
Confidence (math task)					0.081***<br>(0.018)			0.065***<br>(0.021)						
Fetal growth controls	No	No	No	No	No	Yes	Yes	Yes	No	No	No	No	No	No
Neonatal SES controls	No	No	No	No	No	Yes	Yes	Yes	No	No	No	No	No	No
Constant	0.342***<br>(0.033)	0.253***<br>(0.023)	0.220***<br>(0.044)	0.248***<br>(0.045)	-0.105<br>(0.089)	-0.998<br>(1.095)	-0.536<br>(1.125)	-0.606<br>(1.089)	0.237***<br>(0.044)	0.248***<br>(0.043)	0.237***<br>(0.044)	0.247***<br>(0.045)	0.246***<br>(0.044)	0.251***<br>(0.044)
Observations	205	678	205	205	205	175	175	175	204	204	205	205	204	204
R^2	0.005	0.043	0.079	0.102	0.225	0.214	0.228	0.318	0.068	0.096	0.060	0.066	0.054	0.063

Table S3c. Hormones and economic preferences, competitiveness (continues on next page). OLS regressions, standard errors in parentheses. * $p < 0.05$, ** $p < 0.01$, *** $p < 0.005$. . Dep. variable: Competitiveness is a dummy variable equal to 1 if the participant chose the tournament payment scheme and 0 if the participant chose the piece-rate payment scheme. Risk tolerance and all hormone measures except ln(Total T at birth) are standardized to have mean 0 and a standard deviation of 1. Fetal growth controls include birth weight and gestational time Neonatal SES controls were all recorded during pregnancy and are listed in Table S2. Column (19) is based only on those participants that answered all test questions (for understanding of the UG and TG) correctly. Column (20) is based only on those participants for whom both parents report to be Caucasian.

	(15)	(16)	(17)	(18)	(19)	(20)	(21)	(22)	(23)	(24)	(25)	(26)	(27)	(28)
Sample:	All available data				Test Q correct	Caucasian	Women				Men			
Total T at birth		-0.105			-0.063	-0.055	-0.007				-0.165***			
		(0.056)			(0.039)	(0.038)	(0.048)				(0.050)			
Male	0.288***	0.293***	0.203***	0.201***	0.258***	0.248***								
	(0.075)	(0.077)	(0.065)	(0.065)	(0.078)	(0.076)								
Male X Total T at birth														
Bioavailable T at birth								-0.017				-0.197***		
								(0.037)				(0.063)		
Male X Bioavailable T at birth														
Total AE at birth ratio									-0.012				-0.090	
									(0.048)				(0.050)	
Male X Total AE at birth ratio														
Bioavailable AE at birth ratio										-0.006				-0.101
										(0.038)				(0.059)
Male X Bioavailable AE at birth ratio														
ln(Total T at birth)	-0.133*													
	(0.065)													
$(\text{Total T at birth})^2$		0.005												
		(0.013)												
Estradiol at birth (E2)			0.019											
			(0.032)											
Bioavailable E2 at birth				0.020										
				(0.032)										
Risk tolerance														
Puzzles solved (part 1)														
Confidence (math task)														
Fetal growth controls	No	No	No	No	No	No	No	No	No	No	No	No	No	No
Neonatal SES controls	No	No	No	No	No	No	No	No	No	No	No	No	No	No
Constant	0.064	0.211***	0.249***	0.251***	0.232***	0.218***	0.248***	0.248***	0.247***	0.251***	0.538***	0.525***	0.487***	0.480***
	(0.101)	(0.051)	(0.042)	(0.043)	(0.048)	(0.047)	(0.044)	(0.042)	(0.042)	(0.041)	(0.056)	(0.055)	(0.054)	(0.054)
Observations	205	205	211	210	171	172	116	115	116	115	89	89	89	89
R^2	0.068	0.079	0.049	0.048	0.061	0.060	0.000	0.002	0.001	0.000	0.112	0.100	0.037	0.033

Table S3c. Hormones and economic preferences, competitiveness (continued).

	(1)	(2)	(3)	(4)	(5)	(6)	(7)	(8)	(9)	(10)	(11)	(12)	(13)	(14)	(15)	(16)	(17)	(18)	(19)	(20)	(21)	(22)	(23)	(24)	(25)	(26)
Sample:	All available data																Test Q correct	Caucasian	Women				Men			
Total T at birth	0.189*		0.138	0.077	0.092	0.114								0.110			0.088	0.119	0.077				0.190			
	(0.077)		(0.084)	(0.124)	(0.103)	(0.145)								(0.136)			(0.092)	(0.090)	(0.124)				(0.114)			
Male		0.179*	0.255	0.250	0.347	0.352	0.300	0.281	0.322	0.321	0.339*	0.333*	0.239	0.274	0.354*	0.347*	0.244	0.281								
		(0.077)	(0.171)	(0.172)	(0.208)	(0.210)	(0.163)	(0.166)	(0.163)	(0.164)	(0.160)	(0.161)	(0.182)	(0.186)	(0.155)	(0.155)	(0.188)	(0.181)								
Male X Total T at birth				0.114		-0.043																				
				(0.169)		(0.202)																				
Bioavailable T at birth							0.109	0.078												0.078				0.184		
							(0.080)	(0.096)												(0.095)				(0.147)		
Male X Bioavailable T at birth								0.106																		
								(0.175)																		
Total AE at birth ratio									0.085	0.051											0.051				0.111	
									(0.081)	(0.123)											(0.123)				(0.108)	
Male X Total AE at birth ratio										0.060																
										(0.164)																
Bioavailable AE at birth ratio											0.065	0.040										0.040				0.108
											(0.078)	(0.099)										(0.098)				(0.130)
Male X Bioavailable AE at birth ratio												0.068														
												(0.163)														
ln(Total T at birth)													0.221													
													(0.156)													
(Total T at birth)^2														0.009												
														(0.033)												
Estradiol at birth (E2)															0.005											
															(0.076)											
Bioavailable E2 at birth																0.002										
																(0.076)										
Fetal growth controls	No	No	No	No	Yes	Yes	No	No	No	No	No	No	No	No	No	No	No	No	No	No	No	No	No	No	No	No
Neonatal SES controls	No	No	No	No	Yes	Yes	No	No	No	No	No	No	No	No	No	No	No	No	No	No	No	No	No	No	No	No
Constant	-0.048	-0.072	-0.157	-0.178	0.888	1.032	-0.171	-0.178	-0.184	-0.192	-0.187	-0.190	0.106	-0.174	-0.194	-0.187	-0.126	-0.178	-0.178	-0.178	-0.192	-0.190	0.072	0.103	0.129	0.143
	(0.078)	(0.049)	(0.107)	(0.111)	(2.753)	(2.845)	(0.105)	(0.106)	(0.105)	(0.107)	(0.104)	(0.105)	(0.242)	(0.124)	(0.100)	(0.101)	(0.115)	(0.111)	(0.111)	(0.106)	(0.107)	(0.105)	(0.131)	(0.129)	(0.124)	(0.122)
Observations	208	694	208	208	176	176	207	207	208	208	207	207	208	208	215	214	171	173	118	117	118	117	90	90	90	90
R^2	0.029	0.008	0.039	0.041	0.146	0.147	0.034	0.036	0.031	0.032	0.028	0.029	0.036	0.039	0.025	0.024	0.025	0.037	0.003	0.006	0.001	0.001	0.031	0.017	0.012	0.008

Table S3d. Hormones and economic preferences, patience (self-report). OLS regressions, standard errors in parentheses. * $p < 0.05$, ** $p < 0.01$, *** $p < 0.005$. Dep. variable: patience (self-reported). Patience and all hormone measures except ln(Total T at birth) are standardized to have mean 0 and a standard deviation of 1. Fetal growth controls include birth weight and gestational time. Neonatal SES controls were all recorded during pregnancy and are listed in Table S2. Column (17) is based only on those participants that answered all test questions (for understanding of the UG and TG) correctly. Column (18) is based only on those participants for whom both parents report to be Caucasian.

	(1)	(2)	(3)	(4)	(5)	(6)	(7)	(8)	(9)	(10)	(11)	(12)	(13)	(14)	(15)	(16)	(17)	(18)	(19)	(20)	(21)	(22)	(23)	(24)	(25)	(26)
Sample:	All available data																Test Q correct	Caucasian	Women				Men			
Total T at birth	0.068*		0.052	0.032	0.050	0.023								0.060			0.054	0.055	0.032				0.069			
	(0.031)		(0.034)	(0.051)	(0.039)	(0.055)								(0.056)			(0.038)	(0.038)	(0.053)				(0.043)			
Male		0.047	0.079	0.077	0.145	0.138	0.093	0.087	0.086	0.085	0.097	0.094	0.062	0.074	0.140*	0.136*	0.083	0.090								
		(0.034)	(0.070)	(0.070)	(0.079)	(0.080)	(0.067)	(0.068)	(0.066)	(0.066)	(0.065)	(0.065)	(0.074)	(0.076)	(0.064)	(0.064)	(0.078)	(0.076)								
Male X Total T at birth				0.037		0.053																				
				(0.069)		(0.077)																				
Bioavailable T at birth							0.043	0.033												0.033				0.067		
							(0.033)	(0.039)												(0.041)				(0.055)		
Male X Bioavailable T at birth								0.034																		
								(0.071)																		
Total AE at birth ratio									0.065*	0.046											0.046				0.080*	
									(0.033)	(0.050)											(0.053)				(0.040)	
Male X Total AE at birth ratio										0.034																
										(0.066)																
Bioavailable AE at birth ratio											0.056	0.042										0.042				0.082
											(0.032)	(0.040)										(0.042)				(0.049)
Male X Bioavailable AE at birth ratio												0.040														
												(0.066)														
ln(Total T at birth)													0.102													
													(0.064)													
$(\text{Total T at birth})^2$														-0.003												
														(0.013)												
Estradiol at birth (E2)															0.001											
															(0.031)											
Bioavailable E2 at birth																-0.002										
																(0.031)										
Fetal growth controls	No	No	No	No	Yes	Yes	No	No	No	No	No	No	No	No	No	No	No	No	No	No	No	No	No	No	No	No
Neonatal SES controls	No	No	No	No	Yes	Yes	No	No	No	No	No	No	No	No	No	No	No	No	No	No	No	No	No	No	No	No
Constant	0.696***	0.712***	0.662***	0.655***	0.431	0.250	0.659***	0.657***	0.660***	0.655***	0.658***	0.656***	0.787***	0.667***	0.629***	0.634***	0.654***	0.658***	0.655***	0.657***	0.655***	0.656***	0.732***	0.744***	0.740***	0.749***
	(0.032)	(0.022)	(0.044)	(0.045)	(1.048)	(1.082)	(0.043)	(0.043)	(0.043)	(0.044)	(0.042)	(0.042)	(0.099)	(0.051)	(0.041)	(0.042)	(0.048)	(0.047)	(0.048)	(0.045)	(0.046)	(0.045)	(0.049)	(0.049)	(0.046)	(0.046)
Observations	208	695	208	208	176	176	207	207	208	208	207	207	208	208	215	214	171	173	118	117	118	117	90	90	90	90
R^2	0.022	0.003	0.028	0.030	0.226	0.228	0.024	0.025	0.036	0.037	0.031	0.033	0.030	0.029	0.022	0.021	0.030	0.031	0.003	0.005	0.006	0.008	0.028	0.016	0.043	0.031

Table S3e. Hormones and economic preferences, trust. OLS regressions, standard errors in parentheses. * $p < 0.05$, ** $p < 0.01$, *** $p < 0.005$. Dep. variable: Trust is a dummy variable equal to 1 if the participant chose to transfer as a first mover in the TG and 0 otherwise. All hormone measures except ln(Total T at birth) are standardized to have mean 0 and a standard deviation of 1. Fetal growth controls include birth weight and gestational time. Neonatal SES controls were all recorded during pregnancy and are listed in Table S2. Column (17) is based only on those participants that answered all test questions (for understanding of the UG and TG) correctly. Column (18) is based only on those participants for whom both parents report to be Caucasian.

	(1)	(2)	(3)	(4)	(5)	(6)	(7)	(8)	(9)	(10)	(11)	(12)	(13)	(14)	(15)	(16)	(17)	(18)	(19)	(20)	(21)	(22)	(23)	(24)	(25)	(26)
Sample:	All available data																Test Q correct	Caucasia n	Women				Men			
Total T at birth	0.017<br>(0.070)		0.003<br>(0.077)	-0.097<br>(0.113)	-0.005<br>(0.090)	-0.055<br>(0.127)								-0.082<br>(0.125)			0.017<br>(0.075)	0.014<br>(0.087)	-0.097<br>(0.098)				0.089<br>(0.121)			
Male		-0.025<br>(0.077)	0.069<br>(0.157)	0.061<br>(0.157)	0.123<br>(0.182)	0.112<br>(0.184)	0.069<br>(0.150)	0.050<br>(0.153)	0.041<br>(0.149)	0.042<br>(0.150)	0.055<br>(0.146)	0.057<br>(0.147)	0.097<br>(0.167)	0.127<br>(0.171)	0.059<br>(0.141)	0.061<br>(0.141)	-0.117<br>(0.153)	0.087<br>(0.175)								
Male X Total T at birth				0.186<br>(0.154)		0.098<br>(0.177)																				
Bioavailable T at birth							0.012<br>(0.073)	-0.020<br>(0.088)												-0.020<br>(0.076)				0.088<br>(0.155)		
Male X Bioavailable T at birth								0.108<br>(0.160)																		
Total AE at birth ratio									0.054<br>(0.074)	0.068<br>(0.113)											0.068<br>(0.097)				0.044<br>(0.114)	
Male X Total AE at birth ratio										-0.024<br>(0.150)																
Bioavailable AE at birth ratio											0.058<br>(0.072)	0.064<br>(0.090)										0.064<br>(0.078)				0.048<br>(0.137)
Male X Bioavailable AE at birth ratio												-0.016<br>(0.149)														
ln(Total T at birth)													-0.041<br>(0.143)													
(Total T at birth)^2														0.026<br>(0.030)												
Estradiol at birth (E2)															-0.052<br>(0.069)											
Bioavailable E2 at birth																-0.049<br>(0.069)										
Fetal growth controls	No	No	No	No	Yes	Yes	No	No	No	No	No	No	No	No	No	No	No	No	No	No	No	No	No	No	No	No
Neonatal SES controls	No	No	No	No	Yes	Yes	No	No	No	No	No	No	No	No	No	No	No	No	No	No	No	No	No	No	No	No
Constant	-0.069<br>(0.071)	0.010<br>(0.049)	-0.098<br>(0.098)	-0.132<br>(0.102)	-3.637<br>(2.414)	-3.969<br>(2.493)	-0.101<br>(0.096)	-0.108<br>(0.097)	-0.087<br>(0.096)	-0.083<br>(0.098)	-0.095<br>(0.095)	-0.094<br>(0.096)	-0.157<br>(0.222)	-0.148<br>(0.114)	-0.074<br>(0.091)	-0.077<br>(0.092)	0.002<br>(0.094)	-0.141<br>(0.107)	-0.132<br>(0.088)	-0.108<br>(0.084)	-0.083<br>(0.085)	-0.094<br>(0.083)	-0.071<br>(0.139)	-0.057<br>(0.136)	-0.042<br>(0.131)	-0.037<br>(0.129)
Observations	208	694	208	208	176	176	207	207	208	208	207	207	208	208	215	214	171	173	118	117	118	117	90	90	90	90
R^2	0.000	0.000	0.001	0.008	0.184	0.186	0.002	0.004	0.004	0.004	0.005	0.005	0.002	0.005	0.003	0.003	0.004	0.002	0.008	0.001	0.004	0.006	0.006	0.004	0.002	0.001

Table S3f. Hormones and economic preferences, positive reciprocity. OLS regressions, standard errors in parentheses. * $p < 0.05$, ** $p < 0.01$, *** $p < 0.005$. Dep. variable: Positive reciprocity is measured as the amount of money returned in the TG (if the first mover would trust, i.e. measured using the strategy method). Positive reciprocity and all hormone measures except ln(Total T at birth) are standardized to have mean 0 and a standard deviation of 1. Fetal growth controls include birth weight and gestational time. Neonatal SES controls were all recorded during pregnancy and are listed in Table S2. Column (17) is based only on those participants that answered all test questions (for understanding of the UG and TG) correctly. Column (18) is based only on those participants for whom both parents report to be Caucasian.

	(1)	(2)	(3)	(4)	(5)	(6)	(7)	(8)	(9)	(10)	(11)	(12)	(13)	(14)	(15)	(16)	(17)	(18)	(19)	(20)	(21)	(22)	(23)	(24)	(25)	(26)
Sample:	All available data																Test Q correct	Caucasia n	Women				Men			
Total T at birth	0.047		-0.010	-0.107	-0.070	-0.243								-0.099			-0.019	-0.062	-0.107				0.075			
	(0.071)		(0.077)	(0.112)	(0.092)	(0.128)								(0.125)			(0.073)	(0.082)	(0.107)				(0.111)			
Male		0.270***	0.286	0.278	0.366*	0.330	0.308*	0.292	0.273	0.274	0.295*	0.302*	0.345*	0.348*	0.343*	0.347*	0.132	0.328*								
		(0.078)	(0.156)	(0.156)	(0.184)	(0.184)	(0.149)	(0.152)	(0.149)	(0.149)	(0.145)	(0.146)	(0.166)	(0.171)	(0.142)	(0.142)	(0.148)	(0.166)								
Male X Total T at birth				0.182		0.336																				
				(0.154)		(0.177)																				
Bioavailable T at birth							-0.046	-0.073												-0.073				0.020		
							(0.072)	(0.086)												(0.082)				(0.141)		
Male X Bioavailable T at birth								0.092																		
								(0.159)																		
Total AE at birth ratio									0.008	0.053											0.053				-0.030	
									(0.075)	(0.111)											(0.106)				(0.107)	
Male X Total AE at birth ratio										-0.084																
										(0.150)																
Bioavailable AE at birth ratio											-0.038	-0.006										-0.006				-0.096
											(0.071)	(0.088)										(0.085)				(0.126)
Male X Bioavailable AE at birth ratio												-0.089														
												(0.149)														
ln(Total T at birth)													-0.113													
													(0.143)													
(Total T at birth)^2														0.027												
														(0.030)												
Estradiol at birth (E2)															-0.053											
															(0.071)											
Bioavailable E2 at birth																-0.052										
																(0.071)										
Fetal growth controls	No	No	No	No	Yes	Yes	No	No	No	No	No	No	No	No	No	No	No	No	No	No	No	No	No	No	No	No
Neonatal SES controls	No	No	No	No	Yes	Yes	No	No	No	No	No	No	No	No	No	No	No	No	No	No	No	No	No	No	No	No
Constant	-0.077	-0.111*	-0.201*	-0.235*	-0.633	-1.858	-0.213*	-0.219*	-0.196*	-0.185	-0.208*	-0.204*	-0.357	-0.255*	-0.238*	-0.242*	-0.211*	-0.177	-0.235*	-0.219*	-0.185	-0.204*	0.043	0.073	0.089	0.099
	(0.071)	(0.050)	(0.098)	(0.102)	(2.446)	(2.508)	(0.097)	(0.097)	(0.096)	(0.098)	(0.096)	(0.096)	(0.223)	(0.115)	(0.093)	(0.093)	(0.091)	(0.103)	(0.098)	(0.093)	(0.094)	(0.092)	(0.124)	(0.122)	(0.118)	(0.115)
Observations	200	663	200	200	170	170	199	199	200	200	199	199	200	200	206	205	171	168	112	111	112	111	88	88	88	88
R^2	0.002	0.018	0.019	0.026	0.170	0.191	0.021	0.023	0.019	0.020	0.021	0.023	0.022	0.023	0.029	0.030	0.005	0.023	0.009	0.007	0.002	0.000	0.005	0.000	0.001	0.007

Table S3g. Hormones and economic preferences, negative reciprocity. OLS regressions, standard errors in parentheses. * $p < 0.05$, ** $p < 0.01$, *** $p < 0.005$. Dep. variable: Negative reciprocity is measured as the indicated MAO in the UG. Negative reciprocity and all hormone measures except ln(Total T at birth) are standardized to have mean 0 and a standard deviation of 1. Fetal growth controls include birth weight and gestational time. Neonatal SES controls were all recorded during pregnancy and are listed in Table S2. Column (17) is based only on those participants that answered all test questions (for understanding of the UG and TG) correctly. Column (18) is based only on those participants for whom both parents report to be Caucasian.

	(1)	(2)	(3)	(4)	(5)	(6)	(7)	(8)	(9)	(10)	(11)	(12)	(13)	(14)	(15)	(16)	(17)	(18)	(19)	(20)	(21)	(22)	(23)	(24)	(25)	(26)
Sample:	All available data																Test Q correct	Caucasian	Women				Men			
Total T at birth	0.070		0.057	0.155	0.071	0.167								0.099			0.009	0.042	0.155				-0.029			
	(0.066)		(0.073)	(0.106)	(0.086)	(0.122)								(0.118)			(0.080)	(0.076)	(0.100)				(0.106)			
Male		0.139	0.063	0.070	0.107	0.127	0.072	0.075	0.089	0.088	0.095	0.084	0.026	0.034	0.070	0.068	0.205	0.153								
		(0.079)	(0.147)	(0.147)	(0.173)	(0.174)	(0.141)	(0.143)	(0.140)	(0.141)	(0.137)	(0.138)	(0.157)	(0.161)	(0.133)	(0.134)	(0.162)	(0.154)								
Male X Total T at birth				-0.184		-0.187																				
				(0.145)		(0.167)																				
Bioavailable T at birth							0.063	0.068												0.068				0.051		
							(0.068)	(0.081)												(0.077)				(0.134)		
Male X Bioavailable T at birth								-0.016																		
								(0.150)																		
Total AE at birth ratio									0.038	0.003											0.003				0.068	
									(0.071)	(0.105)											(0.100)				(0.102)	
Male X Total AE at birth ratio										0.064																
										(0.142)																
Bioavailable AE at birth ratio											0.035	-0.014										-0.014				0.124
											(0.067)	(0.084)										(0.080)				(0.119)
Male X Bioavailable AE at birth ratio												0.138														
												(0.140)														
ln(Total T at birth)													0.141													
													(0.135)													
(Total T at birth)^2														-0.013												
														(0.028)												
Estradiol at birth (E2)															0.035											
															(0.067)											
Bioavailable E2 at birth																0.033										
																(0.067)										
Fetal growth controls	No	No	No	No	Yes	Yes	No	No	No	No	No	No	No	No	No	No	No	No	No	No	No	No	No	No	No	No
Neonatal SES controls	No	No	No	No	Yes	Yes	No	No	No	No	No	No	No	No	No	No	No	No	No	No	No	No	No	No	No	No
Constant	-0.013	-0.057	-0.041	-0.007	-1.101	-0.420	-0.044	-0.042	-0.052	-0.060	-0.053	-0.060	0.139	-0.016	-0.038	-0.036	-0.025	-0.058	-0.007	-0.042	-0.060	-0.060	0.063	0.032	0.028	0.024
	(0.067)	(0.051)	(0.093)	(0.096)	(2.297)	(2.375)	(0.091)	(0.092)	(0.091)	(0.093)	(0.091)	(0.091)	(0.210)	(0.108)	(0.087)	(0.088)	(0.099)	(0.096)	(0.091)	(0.087)	(0.088)	(0.087)	(0.119)	(0.116)	(0.112)	(0.110)
Observations	200	661	200	200	170	170	199	199	200	200	199	199	200	200	206	205	171	168	112	111	112	111	88	88	88	88
R^2	0.006	0.005	0.007	0.015	0.158	0.166	0.007	0.008	0.005	0.006	0.005	0.009	0.009	0.008	0.003	0.003	0.012	0.012	0.021	0.007	0.000	0.000	0.001	0.002	0.005	0.013

Table S3h. Hormones and economic preferences, dishonesty. OLS regressions, standard errors in parentheses. * $p < 0.05$, ** $p < 0.01$, *** $p < 0.005$. Dep. variable: Dishonesty is measured as the number of heads reported in the coin tossing task. Dishonesty and all hormone measures except ln(Total T at birth) are standardized to have mean 0 and a standard deviation of 1. Fetal growth controls include birth weight and gestational time. Neonatal SES controls were all recorded during pregnancy and are listed in Table S2. Column (17) is based only on those participants that answered all test questions (for understanding of the UG and TG) correctly. Column (18) is based only on those participants for whom both parents report to be Caucasian.

	(1)	(2)	(3)	(4)	(5)	(6)	(7)	(8)	(9)	(10)	(11)	(12)	(13)	(14)	(15)	(16)	(17)	(18)	(19)
Sample:	All available data											Test Q correct		Caucasian		Women		Men	
2D:4D (right hand)	-0.050	0.008	0.038	0.006						-0.002		-0.011		-0.022		0.038		-0.032	
	(0.042)	(0.041)	(0.055)	(0.061)						(0.041)		(0.047)		(0.045)		(0.051)		(0.069)	
2D:4D (left hand)					-0.033	0.024	0.036	0.026			0.004		0.004		0.021		0.036		0.010
					(0.042)	(0.041)	(0.057)	(0.064)			(0.042)		(0.047)		(0.046)		(0.053)		(0.066)
Male		0.521***	0.516***	0.503***		0.522***	0.521***	0.509***	0.522***	0.511***	0.501***	0.549***	0.550***	0.475***	0.487***				
		(0.084)	(0.084)	(0.097)		(0.084)	(0.084)	(0.097)	(0.084)	(0.084)	(0.084)	(0.095)	(0.095)	(0.091)	(0.091)				
Male X 2D:4D (right hand)			-0.070	-0.089															
			(0.084)	(0.097)															
Male X 2D:4D (left hand)							-0.025	-0.076											
							(0.083)	(0.099)											
2D:4D (average)									0.020										
									(0.042)										
(2D:4D (right hand))^2										0.057*									
										(0.026)									
(2D:4D (left hand))^2											0.053*								
											(0.023)								
Fetal growth controls	No	No	No	Yes	No	No	No	Yes	No	No	No	No	No	No	No	No	No	No	No
Neonatal SES controls	No	No	No	Yes	No	No	No	Yes	No	No	No	No	No	No	No	No	No	No	No
Constant	0.023	-0.193***	-0.198***	-1.662	0.021	-0.195***	-0.197***	-1.533	-0.195***	-0.246***	-0.239***	-0.165**	-0.167**	-0.203***	-0.213***	-0.198***	-0.197***	0.317***	0.324***
	(0.041)	(0.053)	(0.054)	(1.311)	(0.042)	(0.053)	(0.054)	(1.319)	(0.053)	(0.058)	(0.056)	(0.061)	(0.061)	(0.057)	(0.057)	(0.050)	(0.050)	(0.071)	(0.070)
Observations	597	597	597	496	595	595	595	494	595	597	595	456	454	505	503	350	349	247	246
R^2	0.002	0.063	0.064	0.099	0.001	0.062	0.063	0.096	0.062	0.071	0.071	0.075	0.073	0.057	0.055	0.002	0.001	0.001	0.000

Table S4a. 2D:4D and economic preferences, risk tolerance (incentivized measure). OLS regressions, standard errors in parentheses. * $p < 0.05$, ** $p < 0.01$, *** $p < 0.005$. Dep. variable: risk tolerance (lottery chosen). Risk tolerance and digit ratios (2D:4D) are standardized to have mean 0 and a standard deviation of 1. Fetal growth controls include birth weight and gestational time. Neonatal SES controls were all recorded during pregnancy and are listed in Table S2. Columns (12) and (13) are based only on those participants that answered all test questions (for understanding of the UG and TG) correctly. Columns (14) and (15) are based only on those participants for whom both parents report to be Caucasian.

	(1)	(2)	(3)	(4)	(5)	(6)	(7)	(8)	(9)	(10)	(11)	(12)	(13)	(14)	(15)	(16)	(17)	(18)	(19)
Sample:	All available data											Test Q correct		Caucasian		Women		Men	
2D:4D (right hand)	-0.088*	-0.038	-0.014	0.004						-0.035		-0.031		-0.016		-0.014		-0.071	
	(0.042)	(0.042)	(0.056)	(0.063)						(0.042)		(0.046)		(0.046)		(0.052)		(0.069)	
2D:4D (left hand)					-0.065	-0.015	0.003	0.014			-0.014		-0.023		-0.015		0.003		-0.034
					(0.042)	(0.042)	(0.058)	(0.065)			(0.043)		(0.046)		(0.046)		(0.054)		(0.066)
Male		0.447***	0.444***	0.516***		0.462***	0.461***	0.518***	0.454***	0.449***	0.463***	0.370***	0.377***	0.491***	0.496***				
		(0.085)	(0.085)	(0.098)		(0.085)	(0.085)	(0.098)	(0.086)	(0.085)	(0.086)	(0.092)	(0.093)	(0.093)	(0.093)				
Male X 2D:4D (right hand)			-0.057	-0.001															
			(0.085)	(0.099)															
Male X 2D:4D (left hand)							-0.037	-0.022											
							(0.084)	(0.100)											
2D:4D (average)									-0.029										
									(0.042)										
(2D:4D (right hand))^2										-0.014									
										(0.026)									
(2D:4D (left hand))^2											-0.002								
											(0.024)								
Fetal growth controls	No	No	No	Yes	No	No	No	Yes	No	No	No	No	No	No	No	No	No	No	No
Neonatal SES controls	No	No	No	Yes	No	No	No	Yes	No	No	No	No	No	No	No	No	No	No	No
Constant	-0.014	-0.204***	-0.209***	3.225*	-0.016	-0.212***	-0.216***	3.196*	-0.209***	-0.191***	-0.210***	-0.169***	-0.174***	-0.217***	-0.222***	-0.209***	-0.216***	0.235***	0.245***
	(0.042)	(0.055)	(0.055)	(1.400)	(0.042)	(0.055)	(0.055)	(1.409)	(0.055)	(0.060)	(0.058)	(0.059)	(0.059)	(0.059)	(0.059)	(0.051)	(0.052)	(0.070)	(0.070)
Observations	560	560	560	468	558	558	558	466	558	560	558	456	454	472	470	322	321	238	237
R^2	0.008	0.055	0.056	0.107	0.004	0.055	0.055	0.107	0.055	0.055	0.055	0.040	0.041	0.061	0.062	0.000	0.000	0.004	0.001

Table S4b. 2D:4D and economic preferences, risk tolerance (self-report). OLS regressions, standard errors in parentheses. * $p < 0.05$, ** $p < 0.01$, *** $p < 0.005$. Dep. variable: risk tolerance (self-report). Risk tolerance and digit ratios (2D:4D) are standardized to have mean 0 and a standard deviation of 1. Fetal growth controls include birth weight and gestational time. Neonatal SES controls were all recorded during pregnancy and are listed in Table S2. Columns (12) and (13) are based only on those participants that answered all test questions (for understanding of the UG and TG) correctly. Columns (14) and (15) are based only on those participants for whom both parents report to be Caucasian.

	(1)	(2)	(3)	(4)	(5)	(6)	(7)	(8)	(9)	(10)	(11)	(12)	(13)
Sample:	All available data												
2D:4D (right hand)	-0.005<br>(0.021)	0.018<br>(0.021)	0.016<br>(0.028)	0.016<br>(0.026)	0.013<br>(0.029)							0.015<br>(0.021)	
2D:4D (left hand)						-0.017<br>(0.020)	0.005<br>(0.021)	0.000<br>(0.029)	-0.001<br>(0.027)	-0.010<br>(0.030)			0.000<br>(0.021)
Male		0.205***<br>(0.041)	0.205***<br>(0.041)	0.090*<br>(0.041)	0.094*<br>(0.047)		0.196***<br>(0.041)	0.196***<br>(0.042)	0.084*<br>(0.041)	0.090<br>(0.047)	0.201***<br>(0.042)	0.203***<br>(0.041)	0.192***<br>(0.042)
Male X 2D:4D (right hand)			0.005<br>(0.041)	-0.015<br>(0.039)	-0.015<br>(0.045)								
Male X 2D:4D (left hand)								0.009<br>(0.041)	-0.002<br>(0.038)	0.016<br>(0.045)			
2D:4D (average)											0.013<br>(0.021)		
(2D:4D (right hand))^2												0.014<br>(0.013)	
(2D:4D (left hand))^2													0.011<br>(0.011)
Risk tolerance				0.071***<br>(0.019)	0.066***<br>(0.022)				0.070***<br>(0.019)	0.065***<br>(0.022)			
Puzzles solved (part 1)				0.010*<br>(0.005)	0.011<br>(0.005)				0.010*<br>(0.005)	0.011<br>(0.005)			
Confidence (math task)				0.057***<br>(0.010)	0.044***<br>(0.012)				0.057***<br>(0.010)	0.044***<br>(0.012)			
Fetal growth controls	No	No	No	No	Yes	No	No	No	No	Yes	No	No	No
Neonatal SES controls	No	No	No	No	Yes	No	No	No	No	Yes	No	No	No
Constant	0.348***<br>(0.020)	0.259***<br>(0.027)	0.260***<br>(0.027)	-0.092<br>(0.055)	-0.559<br>(0.644)	0.347***<br>(0.020)	0.263***<br>(0.027)	0.264***<br>(0.027)	-0.089<br>(0.055)	-0.546<br>(0.647)	0.261***<br>(0.027)	0.246***<br>(0.029)	0.253***<br>(0.028)
Observations	549	549	549	548	458	547	547	547	546	456	547	549	547
R^2	0.000	0.043	0.043	0.172	0.214	0.001	0.041	0.041	0.170	0.212	0.041	0.045	0.043

Table S4c. 2D:4D and economic preferences, competitiveness (continues on next page). OLS regressions, standard errors in parentheses. * $p < 0.05$, ** $p < 0.01$, *** $p < 0.005$. Dep. variable: Competitiveness is a dummy variable equal to 1 if the participant chose the tournament payment scheme and 0 if the participant chose the piece-rate payment scheme. Risk tolerance and digit ratios (2D:4D) are standardized to have mean 0 and a standard deviation of 1. Fetal growth controls include birth weight and gestational time. Neonatal SES controls were all recorded during pregnancy and are listed in Table S2. Columns (14) and (15) are based only on those participants that answered all test questions (for understanding of the UG and TG) correctly. Columns (16) and (17) are based only on those participants for whom both parents report to be Caucasian.

	(14)	(15)	(16)	(17)	(18)	(19)	(20)	(21)
Sample:	Test Q correct		Caucasian		Women		Men	
2D:4D (right hand)	0.013		0.015		0.016		0.021	
	(0.023)		(0.022)		(0.026)		(0.033)	
2D:4D (left hand)		0.002		-0.004		0.000		0.009
		(0.023)		(0.023)		(0.027)		(0.032)
Male	0.213***	0.205***	0.206***	0.193***				
	(0.046)	(0.046)	(0.045)	(0.045)				
Male X 2D:4D (right hand)								
Male X 2D:4D (left hand)								
2D:4D (average)								
$(2D{:}4D \text{ (right hand)})^2$								
$(2D{:}4D \text{ (left hand)})^2$								
Risk tolerance								
Puzzles solved (part 1)								
Confidence (math task)								
Fetal growth controls	No	No	No	No	No	No	No	No
Neonatal SES controls	No	No	No	No	No	No	No	No
Constant	0.265***	0.268***	0.254***	0.259***	0.260***	0.264***	0.465***	0.460***
	(0.029)	(0.030)	(0.029)	(0.029)	(0.025)	(0.026)	(0.034)	(0.034)
Observations	456	454	463	461	312	311	237	236
R^2	0.046	0.044	0.044	0.041	0.001	0.000	0.002	0.000

Table S4c. 2D:4D and economic preferences, competitiveness (continued).

	(1)	(2)	(3)	(4)	(5)	(6)	(7)	(8)	(9)	(10)	(11)	(12)	(13)	(14)	(15)	(16)	(17)	(18)	(19)
Sample:	All available data											Test Q correct		Caucasian		Women		Men	
2D:4D (right hand)	-0.026	0.001	0.015	0.020						0.007		-0.001		-0.015		0.015		-0.017	
	(0.043)	(0.044)	(0.058)	(0.064)						(0.044)		(0.049)		(0.047)		(0.057)		(0.069)	
2D:4D (left hand)					-0.050	-0.023	-0.018	-0.040			-0.016		-0.004		-0.057		-0.018		-0.027
					(0.042)	(0.043)	(0.060)	(0.066)			(0.044)		(0.049)		(0.047)		(0.058)		(0.065)
Male		0.250^{***}	0.249^{**}	0.251^{*}		0.253^{***}	0.253^{***}	0.241^{*}	0.256^{***}	0.254^{***}	0.260^{***}	0.189	0.204^{*}	0.250^{**}	0.246^{**}				
		(0.088)	(0.089)	(0.099)		(0.088)	(0.088)	(0.099)	(0.089)	(0.088)	(0.089)	(0.098)	(0.099)	(0.095)	(0.095)				
Male X 2D:4D (right hand)			-0.032	-0.009															
			(0.088)	(0.101)															
Male X 2D:4D (left hand)							-0.008	0.013											
							(0.087)	(0.101)											
2D:4D (average)									-0.014										
									(0.044)										
$(\text{2D:4D (right hand)})^2$										-0.024									
										(0.027)									
$(\text{2D:4D (left hand)})^2$											-0.017								
											(0.024)								
Fetal growth controls	No	No	No	Yes	No	No	No	Yes	No	No	No	No	No	No	No	No	No	No	No
Neonatal SES controls	No	No	No	Yes	No	No	No	Yes	No	No	No	No	No	No	No	No	No	No	No
Constant	-0.022	-0.128^{*}	-0.131^{*}	0.429	-0.021	-0.128^{*}	-0.129^{*}	0.184	-0.130^{*}	-0.106	-0.114	-0.091	-0.096	-0.143^{*}	-0.139^{*}	-0.131^{*}	-0.129^{*}	0.117	0.124
	(0.043)	(0.057)	(0.057)	(1.424)	(0.043)	(0.057)	(0.057)	(1.422)	(0.057)	(0.062)	(0.060)	(0.063)	(0.063)	(0.061)	(0.061)	(0.056)	(0.056)	(0.070)	(0.069)
Observations	560	560	560	468	558	558	558	466	558	560	558	456	454	472	470	322	321	238	237
R^2	0.001	0.015	0.015	0.084	0.003	0.017	0.017	0.087	0.017	0.016	0.018	0.009	0.010	0.016	0.021	0.000	0.000	0.000	0.001

Table S4d. 2D:4D and economic preferences, patience (self-report). OLS regressions, standard errors in parentheses. * $p < 0.05$, ** $p < 0.01$, *** $p < 0.005$. Dep. variable: patience (self-reported). Patience and digit ratios (2D:4D) are standardized to have mean 0 and a standard deviation of 1. Fetal growth controls include birth weight and gestational time. Neonatal SES controls were all recorded during pregnancy and are listed in Table S2. Columns (12) and (13) are based only on those participants that answered all test questions (for understanding of the UG and TG) correctly. Columns (14) and (15) are based only on those participants for whom both parents report to be Caucasian.

	(1)	(2)	(3)	(4)	(5)	(6)	(7)	(8)	(9)	(10)	(11)	(12)	(13)	(14)	(15)	(16)	(17)	(18)	(19)
Sample:	All available data											Test Q correct		Caucasian		Women		Men	
2D:4D (right hand)	0.007<br>(0.019)	0.013<br>(0.019)	-0.000<br>(0.025)	-0.001<br>(0.028)						0.016<br>(0.019)		0.015<br>(0.022)		0.014<br>(0.020)		-0.000<br>(0.026)		0.031<br>(0.028)	
2D:4D (left hand)					-0.003<br>(0.019)	0.003<br>(0.019)	-0.030<br>(0.026)	-0.029<br>(0.029)			0.002<br>(0.020)		0.010<br>(0.022)		0.004<br>(0.021)		-0.030<br>(0.027)		0.040<br>(0.026)
Male		0.061<br>(0.039)	0.062<br>(0.039)	0.047<br>(0.044)		0.056<br>(0.039)	0.058<br>(0.039)	0.044<br>(0.044)	0.059<br>(0.039)	0.062<br>(0.039)	0.055<br>(0.039)	0.070<br>(0.043)	0.069<br>(0.043)	0.051<br>(0.041)	0.046<br>(0.041)				
Male X 2D:4D (right hand)			0.032<br>(0.039)	0.027<br>(0.044)															
Male X 2D:4D (left hand)							0.070<br>(0.038)	0.065<br>(0.045)											
2D:4D (average)									0.010<br>(0.019)										
(2D:4D (right hand))^2										-0.013<br>(0.012)									
(2D:4D (left hand))^2											0.003<br>(0.011)								
Fetal growth controls	No	No	No	Yes	No	No	No	Yes	No	No	No	No	No	No	No	No	No	No	No
Neonatal SES controls	No	No	No	Yes	No	No	No	Yes	No	No	No	No	No	No	No	No	No	No	No
Constant	0.734***<br>(0.019)	0.708***<br>(0.025)	0.711***<br>(0.025)	0.701<br>(0.629)	0.733***<br>(0.019)	0.710***<br>(0.025)	0.716***<br>(0.025)	0.632<br>(0.631)	0.708***<br>(0.025)	0.720***<br>(0.027)	0.707***<br>(0.027)	0.707***<br>(0.028)	0.706***<br>(0.028)	0.737***<br>(0.026)	0.738***<br>(0.026)	0.711***<br>(0.026)	0.716***<br>(0.026)	0.774***<br>(0.028)	0.775***<br>(0.028)
Observations	561	561	561	468	559	559	559	466	559	561	559	456	454	472	470	322	321	239	238
R^2	0.000	0.005	0.006	0.061	0.000	0.004	0.010	0.064	0.004	0.007	0.004	0.006	0.006	0.004	0.003	0.000	0.004	0.005	0.010

Table S4e. 2D:4D and economic preferences, trust. OLS regressions, standard errors in parentheses. * $p < 0.05$, ** $p < 0.01$, *** $p < 0.005$. Dep. variable: Trust is a dummy variable equal to 1 if the participant chose to transfer as a first mover in the TG and 0 otherwise. Digit ratios (2D:4D) are standardized to have mean 0 and a standard deviation of 1. Fetal growth controls include birth weight and gestational time. Neonatal SES controls were all recorded during pregnancy and are listed in Table S2. Columns (12) and (13) are based only on those participants that answered all test questions (for understanding of the UG and TG) correctly. Columns (14) and (15) are based only on those participants for whom both parents report to be Caucasian.

	(1)	(2)	(3)	(4)	(5)	(6)	(7)	(8)	(9)	(10)	(11)	(12)	(13)	(14)	(15)	(16)	(17)	(18)	(19)
Sample:	All available data											Test Q correct		Caucasian		Women		Men	
2D:4D (right hand)	0.045	0.045	0.007	-0.000						0.048		0.034		0.038		0.007		0.094	
	(0.042)	(0.043)	(0.057)	(0.063)						(0.043)		(0.046)		(0.044)		(0.048)		(0.077)	
2D:4D (left hand)					0.064	0.065	0.021	0.030			0.058		0.057		0.055		0.021		0.113
					(0.041)	(0.043)	(0.059)	(0.065)			(0.044)		(0.046)		(0.045)		(0.049)		(0.073)
Male		-0.005	-0.000	-0.050		0.004	0.007	-0.038	0.006	-0.003	-0.003	-0.054	-0.041	0.024	0.031				
		(0.087)	(0.087)	(0.099)		(0.087)	(0.087)	(0.099)	(0.087)	(0.087)	(0.087)	(0.093)	(0.093)	(0.090)	(0.090)				
Male X 2D:4D (right hand)			0.087	0.129															
			(0.086)	(0.100)															
Male X 2D:4D (left hand)							0.092	0.120											
							(0.085)	(0.100)											
2D:4D (average)									0.063										
									(0.043)										
(2D:4D (right hand))^2										-0.014									
										(0.027)									
(2D:4D (left hand))^2											0.016								
											(0.024)								
Fetal growth controls	No	No	No	Yes	No	No	No	Yes	No	No	No	No	No	No	No	No	No	No	No
Neonatal SES controls	No	No	No	Yes	No	No	No	Yes	No	No	No	No	No	No	No	No	No	No	No
Constant	-0.003	-0.001	0.007	-2.250	-0.004	-0.006	0.003	-2.293	-0.007	0.012	-0.019	0.031	0.024	-0.005	-0.010	0.007	0.003	0.007	0.010
	(0.042)	(0.056)	(0.056)	(1.410)	(0.042)	(0.056)	(0.056)	(1.417)	(0.056)	(0.061)	(0.059)	(0.059)	(0.060)	(0.057)	(0.057)	(0.047)	(0.048)	(0.078)	(0.078)
Observations	560	560	560	468	558	558	558	466	558	560	558	456	454	472	470	322	321	238	237
R^2	0.002	0.002	0.004	0.049	0.004	0.004	0.006	0.052	0.004	0.003	0.005	0.003	0.005	0.002	0.003	0.000	0.001	0.006	0.010

Table S4f. 2D:4D and economic preferences, positive reciprocity. OLS regressions, standard errors in parentheses. * $p < 0.05$, ** $p < 0.01$, *** $p < 0.005$. Dep. variable: Positive reciprocity is measured as the amount of money returned in the TG (if the first mover would trust, i.e. measured using the strategy method). Positive reciprocity and digit ratios (2D:4D) are standardized to have mean 0 and a standard deviation of 1. Fetal growth controls include birth weight and gestational time. Neonatal SES controls were all recorded during pregnancy and are listed in Table S2. Columns (12) and (13) are based only on those participants that answered all test questions (for understanding of the UG and TG) correctly. Columns (14) and (15) are based only on those participants for whom both parents report to be Caucasian.

	(1)	(2)	(3)	(4)	(5)	(6)	(7)	(8)	(9)	(10)	(11)	(12)	(13)	(14)	(15)	(16)	(17)	(18)	(19)
Sample:	All available data											Test Q correct		Caucasian		Women		Men	
2D:4D (right hand)	-0.060	-0.034	-0.071	-0.059						-0.043		-0.035		-0.030		-0.071		0.014	
	(0.043)	(0.044)	(0.059)	(0.066)						(0.044)		(0.046)		(0.048)		(0.056)		(0.070)	
2D:4D (left hand)					-0.021	0.009	-0.026	0.058			0.002		-0.023		0.001		-0.026		0.046
					(0.043)	(0.044)	(0.061)	(0.067)			(0.045)		(0.046)		(0.048)		(0.058)		(0.067)
Male		0.252***	0.256***	0.317***		0.273***	0.276***	0.348***	0.263***	0.245**	0.267***	0.204*	0.213*	0.256**	0.273***				
		(0.088)	(0.088)	(0.099)		(0.089)	(0.089)	(0.100)	(0.089)	(0.088)	(0.089)	(0.091)	(0.092)	(0.096)	(0.096)				
Male X 2D:4D (right hand)			0.085	0.004															
			(0.089)	(0.102)															
Male X 2D:4D (left hand)							0.072	-0.073											
							(0.088)	(0.102)											
2D:4D (average)									-0.014										
									(0.044)										
(2D:4D (right hand))^2										0.048									
										(0.028)									
(2D:4D (left hand))^2											0.017								
											(0.024)								
Fetal growth controls	No	No	No	Yes	No	No	No	Yes	No	No	No	No	No	No	No	No	No	No	No
Neonatal SES controls	No	No	No	Yes	No	No	No	Yes	No	No	No	No	No	No	No	No	No	No	No
Constant	0.012	-0.097	-0.090	-0.898	0.012	-0.106	-0.099	-0.855	-0.101	-0.141*	-0.120*	-0.093	-0.097	-0.092	-0.101	-0.090	-0.099	0.166*	0.177*
	(0.043)	(0.057)	(0.058)	(1.433)	(0.043)	(0.057)	(0.058)	(1.442)	(0.058)	(0.062)	(0.061)	(0.058)	(0.059)	(0.062)	(0.062)	(0.055)	(0.055)	(0.071)	(0.071)
Observations	537	537	537	449	535	535	535	447	535	537	535	456	454	452	450	305	304	232	231
R^2	0.004	0.019	0.020	0.093	0.000	0.018	0.019	0.092	0.018	0.024	0.019	0.015	0.015	0.019	0.018	0.005	0.001	0.000	0.002

Table S4g. 2D:4D and economic preferences, negative reciprocity. OLS regressions, standard errors in parentheses. * $p < 0.05$, ** $p < 0.01$, *** $p < 0.005$. Dep. variable: Negative reciprocity is measured as the indicated MAO in the UG. Negative reciprocity and digit ratios (2D:4D) are standardized to have mean 0 and a standard deviation of 1. Fetal growth controls include birth weight and gestational time. Neonatal SES controls were all recorded during pregnancy and are listed in Table S2. Columns (12) and (13) are based only on those participants that answered all test questions (for understanding of the UG and TG) correctly. Columns (14) and (15) are based only on those participants for whom both parents report to be Caucasian.

	(1)	(2)	(3)	(4)	(5)	(6)	(7)	(8)	(9)	(10)	(11)	(12)	(13)	(14)	(15)	(16)	(17)	(18)	(19)
Sample:	All available data											Test Q correct		Caucasian		Women		Men	
2D:4D (right hand)	-0.076	-0.061	-0.015	-0.025						-0.064		-0.074		-0.067		-0.015		-0.116	
	(0.045)	(0.046)	(0.061)	(0.070)						(0.046)		(0.050)		(0.050)		(0.057)		(0.074)	
2D:4D (left hand)					-0.035	-0.018	0.036	0.023			-0.007		-0.039		-0.025		0.036		-0.076
					(0.044)	(0.045)	(0.063)	(0.071)			(0.046)		(0.050)		(0.050)		(0.058)		(0.071)
Male		0.153	0.148	0.171		0.165	0.161	0.183	0.153	0.150	0.176	0.214*	0.223*	0.120	0.129				
		(0.091)	(0.091)	(0.105)		(0.091)	(0.091)	(0.106)	(0.092)	(0.091)	(0.092)	(0.100)	(0.101)	(0.098)	(0.099)				
Male X 2D:4D (right hand)			-0.101	-0.111															
			(0.092)	(0.108)															
Male X 2D:4D (left hand)							-0.113	-0.108											
							(0.090)	(0.109)											
2D:4D (average)									-0.042										
									(0.046)										
(2D:4D (right hand))^2										0.023									
										(0.029)									
(2D:4D (left hand))^2											-0.029								
											(0.025)								
Fetal growth controls	No	No	No	Yes	No	No	No	Yes	No	No	No	No	No	No	No	No	No	No	No
Neonatal SES controls	No	No	No	Yes	No	No	No	Yes	No	No	No	No	No	No	No	No	No	No	No
Constant	0.011	-0.055	-0.063	0.378	0.010	-0.062	-0.072	0.563	-0.057	-0.076	-0.038	-0.020	-0.025	-0.074	-0.082	-0.063	-0.072	0.085	0.089
	(0.044)	(0.059)	(0.059)	(1.520)	(0.044)	(0.059)	(0.060)	(1.528)	(0.059)	(0.064)	(0.063)	(0.064)	(0.065)	(0.064)	(0.064)	(0.055)	(0.056)	(0.074)	(0.075)
Observations	535	535	535	448	533	533	533	446	533	535	533	455	453	450	448	304	303	231	230
R^2	0.005	0.011	0.013	0.054	0.001	0.007	0.010	0.052	0.009	0.012	0.010	0.019	0.015	0.009	0.005	0.000	0.001	0.011	0.005

Table S4h. 2D:4D and economic preferences, dishonesty. OLS regressions, standard errors in parentheses. $^{*}p < 0.05$, $^{**}p < 0.01$, $^{***}p < 0.005$. Dep. variable: Dishonesty is measured as the number of heads reported in the coin tossing task. Dishonesty and digit ratios (2D:4D) are standardized to have mean 0 and a standard deviation of 1. Fetal growth controls include birth weight and gestational time. Neonatal SES controls were all recorded during pregnancy and are listed in Table S2. Columns (12) and (13) are based only on those participants that answered all test questions (for understanding of the UG and TG) correctly. Columns (14) and (15) are based only on those participants for whom both parents report to be Caucasian.

	Point estimate	SE	90% CI		MDE	One-sided *t*-tests *p*-value LB	*p*-value UB
**Total T at birth**							
Risk tolerance	0.084	0.072	-0.035	0.202	0.224	0.000	0.026
Risk tolerance (self-report)	0.028	0.078	-0.100	0.157	0.224	0.001	0.006
Competitiveness	-0.090	0.035	-0.148	-0.032	0.112	0.265	0.000
Patience (self-report)	0.138	0.084	-0.001	0.277	0.224	0.000	0.152
Trust	0.052	0.034	-0.004	0.109	0.112	0.000	0.041
Positive reciprocity	0.003	0.077	-0.124	0.131	0.224	0.002	0.002
Negative reciprocity	-0.010	0.077	-0.137	0.117	0.224	0.003	0.001
Dishonesty	0.057	0.073	-0.063	0.177	0.224	0.000	0.011
**2D:4D (right hand)**							
Risk tolerance	0.008	0.041	-0.060	0.076	0.139	0.000	0.001
Risk tolerance (self-report)	-0.038	0.042	-0.107	0.031	0.139	0.008	0.000
Competitiveness	0.018	0.021	-0.016	0.052	0.070	0.000	0.006
Patience (self-report)	0.001	0.044	-0.071	0.073	0.139	0.001	0.001
Trust	0.013	0.019	-0.018	0.045	0.070	0.000	0.002
Positive reciprocity	0.045	0.043	-0.026	0.115	0.139	0.000	0.014
Negative reciprocity	-0.034	0.044	-0.106	0.039	0.139	0.008	0.000
Dishonesty	-0.061	0.046	-0.136	0.014	0.139	0.042	0.000
**2D:4D (left hand)**							
Risk tolerance	0.024	0.041	-0.044	0.092	0.139	0.000	0.003
Risk tolerance (self-report)	-0.015	0.042	-0.083	0.054	0.139	0.001	0.000
Competitiveness	0.005	0.021	-0.029	0.038	0.070	0.000	0.001
Patience (self-report)	-0.023	0.043	-0.094	0.049	0.139	0.004	0.000
Trust	0.003	0.019	-0.028	0.035	0.070	0.000	0.000
Positive reciprocity	0.065	0.043	-0.005	0.135	0.139	0.000	0.040
Negative reciprocity	0.009	0.044	-0.064	0.081	0.139	0.000	0.002
Dishonesty	-0.018	0.045	-0.092	0.057	0.139	0.004	0.000

Table S5. Equivalence tests for testosterone at birth, 2D:4D and economic preferences. Point estimates, standard errors and 90% CI come from OLS regressions of the preference measure on total T at birth or 2D:4D and a sex dummy. The minimum detectable effect size (MDE) is based on α = 0.05, power = 0.90, and n = 200 for total T at birth and n = 533 for both 2D:4D measures. The reported p-values of the two one-sided t-tests test the null hypothesis that the point estimate is larger (in absolute) value than the negative (LB) and positive (UB) value of the MDE.

							Correcting for 4 hypotheses (all 4 hormone measures)		Correcting for 8 hypotheses (all 8 preference measures)		Correcting for 32 hypotheses (all 32 hormone/preference combinations)	
Preference measure	Hormone measure	Effect size	Standard error	95% CI	95% CI	Uncorrected *p*-value	Bonferroni *q*-value	FDR *q*-value	Bonferroni *q*-value	FDR *q*-value	Bonferroni *q*-value	FDR *q*-value
Competitiveness	Total T	-0.090	0.035	-0.159	-0.021	0.011	0.042	0.042	0.084	0.084	0.337	0.337
Competitiveness	Bioavailable T	-0.070	0.033	-0.135	-0.005	0.036	0.143	0.072	0.286	0.286	1.000	0.517
Trust	Total AE ratio	0.065	0.033	0.000	0.130	0.049	0.194	0.155	0.388	0.388	1.000	0.517

Table S6. *q*-values of statistically significant associations, *p*-values corrected for multiple comparisons.

SM References

Abdellaoui, M., Baillon, A., Placido, L., & Wakker, P. P. (2011). The rich domain of uncertainty: Source functions and their experimental implementation. *American Economic Review*, *101*(2), 695-723.

Abeler, J., Nosenzo, D., & Raymond, C. (2019). Preferences for truth-telling. *Econometrica, 87*(4), 1115-1153.

Almås, I., Cappelen, A. W., Salvanes, K. G., Sørensen, E. Ø., & Tungodden, B. (2015). Willingness to compete: Family matters. *Management Science*, *62*(8), 2149-2162.

Apicella, C. L., Tobolsky, V. A., Marlowe, F. W., & Miller, K. W. (2016). Hadza hunter-gatherer men do not have more masculine digit ratios (2D: 4D). *American Journal of Physical Anthropology*, *159*(2), 223-232.

Armantier, O. (2006). Do wealth differences affect fairness considerations? *International Economic Review*, *47*(2), 391-429.

Balliet, D., Li, N. P., Macfarlan, S. J., & Van Vugt, M. (2011). Sex differences in cooperation: a meta-analytic review of social dilemmas. *Psychological Bulletin, 137*(6), 881.

Berg, J., Dickhaut, J., & McCabe, K. (1995). Trust, reciprocity, and social history. *Games and Economic Behavior*, *10*(1), 122-142.

Bolle, F. (1990). High reward experiments without high expenditure for the experimenter? *Journal of Economic Psychology*, *11*(2), 157-167.

Brañas-Garza, P., Galizzi, M. M., & Nieboer, J. (2018). Experimental and Self-Reported Measures of Risk Taking and Digit Ratio (2d: 4d): Evidence from a Large, Systematic Study. *International Economic Review*, *59*(3), 1131-1157.

Brañas-Garza, P., Kovářík, J., & Neyse, L. (2013). Second-to-fourth digit ratio has a non-monotonic impact on altruism. *PloS One*, *8*(4), e60419.

Buser, T., Geijtenbeek, L., & Plug, E. (2018). Sexual orientation, competitiveness and income. *Journal of Economic Behavior & Organization*, *151*, 191-198.

Charness, G., & Gneezy, U. (2012). Strong evidence for sex differences in risk taking. *Journal of Economic Behavior & Organization, 83*(1), 50-58.

Charness, G., Eckel, C., Gneezy, U., & Kajackaite, A. (2018). Complexity in risk elicitation may affect the conclusions: A demonstration using sex differences. *Journal of Risk and Uncertainty, 56*(1), 1-17.

Croson, R., & Gneezy, U. (2009). Sex differences in preferences. *Journal of Economic Literature*, *47*(2), 448-74.

Dohmen, T., Falk, A., Huffman, D., Sunde, U., Schupp, J., & Wagner, G. G. (2011). Individual risk attitudes: Measurement, determinants, and behavioral consequences. *Journal of the European Economic Association*, *9*(3), 522-550.

Eckel, C. C., & Grossman, P. J. (2002). Sex differences and statistical stereotyping in attitudes toward financial risk. *Evolution and Human Behavior*, *23*(4), 281-295.

Falk, A., Becker, A., Dohmen, T., Enke, B., Huffman, D., & Sunde, U. (2018). Global evidence on economic preferences. *The Quarterly Journal of Economics*, *133*(4), 1645-1692.

Filippin, A., & Crosetto, P. (2016). A reconsideration of sex differences in risk attitudes. *Management Science, 62*(11), 3138-3160.

Fischbacher, U., & Föllmi-Heusi, F. (2013). Lies in disguise—an experimental study on cheating. *Journal of the European Economic Association*, *11*(3), 525-547.

Galizzi, M. M., & Nieboer, J. (2015). Digit ratio (2D: 4D) and altruism: evidence from a large, multi-ethnic sample. *Frontiers in Behavioral Neuroscience, 9,* 41.

Güth, W., Schmittberger, R., & Schwarze, B. (1982). An experimental analysis of ultimatum bargaining. *Journal of Economic Behavior & Organization, 3*(4), 367-388.

Meier, S., & Sprenger, C. D. (2013). Discounting financial literacy: Time preferences and participation in financial education programs. *Journal of Economic Behavior & Organization*, *95*, 159-174.

Nelson, J. A. (2015). Are women really more risk-averse than men? A re-analysis of the literature using expanded methods. *Journal of Economic Surveys, 29*(3), 566-585.

Niederle, M. (2016). Gender, in *Handbook of Experimental Economics, second edition,* Eds. Kagel, J. and Roth, A.E., Princeton University Press, 2016, pp 481-553.

Niederle, M., & Vesterlund, L. (2007). Do women shy away from competition? Do men compete too much?. *The Quarterly Journal of Economics, 122*(3), 1067-1101.

Niederle, M., & Vesterlund, L. (2011). Gender and competition. *Annual Review of Economics, 3*(1), 601-630.

Parslow, E., Ranehill, E., Zethraeus, N., Blomberg, L., von Schoultz, B., & Lindén Hirschberg, A., Johannesson, M. & Dreber, A. (2019). The digit ratio (2D: 4D) and economic preferences: no robust associations in a sample of 330 women. *Journal of the Economic Science Association*, *5*(2), 149-169.

Sapienza, P., Zingales, L., & Maestripieri, D. (2009). Sex differences in financial risk aversion and career choices are affected by testosterone. *Proceedings of the National Academy of Sciences, 106*(36), 15268-15273.

Sutter, M., Kocher, M. G., Glätzle-Rützler, D., & Trautmann, S. T. (2013). Impatience and uncertainty: Experimental decisions predict adolescents' field behavior. *American Economic Review, 103*(1), 510-31.
